# Validating new symptom emergence as a patient-centric outcome measure for PD clinical trials

**DOI:** 10.1016/j.parkreldis.2024.107118

**Published:** 2024-09-10

**Authors:** Haotian Zou, Glenn T. Stebbins, Tanya Simuni, Sheng Luo, Jesse M. Cedarbaum

**Affiliations:** a Department of Biostatistics & Bioinformatics, Duke University, Durham, NC, 27705, USA; b Professor Emeritus, Neurological Sciences, Rush University Medical Center, Chicago, IL, 60612, USA; c Parkinson’s disease and Movement Disorders Center, Northwestern University Feinberg School of Medicine, 710 North Lake Shore Drive, Chicago IL, 60611, USA; d Division of Movement Disorders, Department of Neurology, Yale School of Medicine, New Haven CT 06511, USA

## Abstract

**Introduction:**

Tracking of emergent symptoms (ES) in de novo Parkinson Disease (PD) patients using Parts Ib and II of the MDS-UPDRS rating scale has been proposed as an outcome measure for PD clinical trials, based on observations in the Safety, Tolerability and Efficacy Assessment of Isradipine for PD (STEADY-PD3) clinical trial.

**Methods:**

Individual item-level data was extracted from the SURE-PD3 study (coded as “PD-1018” in the C-path pooled dataset). We sought to confirm the observations made in the STEADY-PD3 dataset by analyzing data from a different Phase 3 clinical trial, the Phase 3 Study of Urate Elevation in Parkinson Disease (SURE-PD3), in which MDS-UPDRS was assessed more frequently than the 12-month intervals in STEADY-PD3, using similar methodology.

**Results:**

We were able to broadly validate results that demonstrated the frequency of ES, lack of impact of the introduction of symptomatic medications, and in the reduction in sample size required to demonstrate slowing of disease progression at a group level compared with the traditional total MDS-UPDRS summed score scoring methods. Counts of ES generally correlated modestly with summed MDS-UPRDS scores, both for the various sub-parts and for the overall scale as well. However, instability of individual item responses, especially during the first 6 months of observation complicated the assessment of the temporal evolution and stability of ES over time in the course of the SURE-PD3 study.

**Conclusion:**

Further validation using data sets with frequent administration of MDS-UPDRS is necessary to assess value of this approach as an outcome measure in PD clinical trials.

## 1. Introduction

Tracking progression in the early, recently diagnosed (“de novo”) PD has proven to be a daunting task. The Movement Disorders Society Unified Parkinson Disease Rating Scale (MDS-UPDRS) [1] has been the most commonly used outcome measure but has a number of limitations. Summed scores from either Parts I, II, III or the total score moves only a fraction of the dynamic range of the scale in the 1–2 year time span of a typical Phase 2 or 3 “disease modification” clinical trial [2,3], and the sensitivity of the scale has been suggested to be inadequate especially in the earliest stages of disability [4,5]. Furthermore, there is an increasing interest in the use of patient-centric, Patient Reported Outcome Measures (PROs) as outcome measures for pivotal trials supporting full approval of new therapeutics for PD.

The slow pace of evolution of the motor and cognitive symptoms of PD presents a challenge monitoring progression of PD. For example, in the Parkinson Progression Markers Initiative (PPMI) data set, the change at one year for Parts 1, 2 or 3 of the MDS-UPDRS averages about 2 % of the total score for each part (based on data in Holden et al. [2]. This moderate degree of change over time has yielded sample size estimates to see a 50 % or 25 % reduction progression as assessed by Parts 1 or 2 of the MDS-UPDRS at 1 and 2 years of 524 and 2088 for Part 1 and 440 and 1752 for Part 2 [3] for clinical trials of agents intended to slow the course of the disease, rather than directly improve symptoms. Thus, there is a need for more robust assessment methods for early, de novo PD. A variety of proposals have emerged to attempt to deal with this problem, as summarized in the procedings of a recent workshop [3] ranging from the development of completely novel scales and the use of digital outcome assessments to the use of sub-components or item subsets from existing scales. It has been recognized that rigourous development and validation of novel measures that reflect lived patient and care partner experience is a rigorous and time-consuming process, as outlined in a recent series of FDA Guidance document [6]. But as novel outcome measures for PD make their way to the fore, adaptation and novel analysis methods for current instruments constitute perhaps the most accessible means to move forward.

Although the MDS-UPDRS rating scale was originally designed as four independent Parts, Zou et al. [7] have provided statistical confirmation that Parts Ib and 2 can function together as a single patient-reported outcome measure (PRO). Tosin et al. [8] published the results of a pilot study in which we explored the potential of a novel way to assess disease progression in de novo PD using Parts 1b and 2 of the MDS-UPDRS as a PRO. In brief, Tosin et al. assessed the frequency of emergent symptoms (ES, as reflected in a score ≥1) that were not present at baseline as reported using this instrument at the 1-year time point in the STEADY-PD3 study (NCT02168842) [9]. It was found that 87 % of study participants endorsed one or more MDS-UPDRS Part 1b or 2 items at 1 year that were not endorsed (scored as “0”) at baseline. The average number of new scale items endorsed at 1 year was 3. Initiation of symptomatic medications during the course of this study did not appear to meaningfully impact the incidence of emergent symptoms. Given the high event rate for ES at 1 year, it was estimated that a sample size of <100 subjects per group would be sufficient to detect a 30 % reduction in the rate of ES, making the ES method for scoring the MDS-UPDRS a promising tool for use in proof-of-concept clinical trials, and perhaps even as a pivotal outcome measure.

However, the study of Tosin et al. [8] suffered from several limitations. Most fundamentally, the results were based on data from Safety, Tolerability, and Efficacy Assessment of Isradipine for PD (STEADY-PD3) a study in which the MDS-UPDRS was collected only annually (Parkinson Study Group 2020; NCT02168842) [10]. This sample was too small to divide into test and validation cohorts. Thus, several additional questions were left unanswered in the pilot study. These questions need to be addressed in order to determine if the ES tracking method provides a valid assessment of progression in de novo PD. We therefore decided to attempt to replicate and extend the findings in our original report using data from the similarly-designed Phase 3 Study of Urate Elevation in Parkinson Disease (SURE-PD3) study (Parkinson Study Group 2021; NCT02642393) [9]. It was believed that this would constitute a convenient resource for conducting our investigations, as this study also used the MDS-UPDRS as an outcome measure, and was conducted in a similar manner to STEADY-PD3, upon which our original publication was based. In SURE-PD3, which had a total N of 298 randomized and 273 completed subjects, the MDS-UPDRS was administered every 3 months instead of at annual intervals as in STEADY-PD3, which could also provide an opportunity not only to replicate our findings, but also answer additional questions.

We therefore sought to replicate the results of Tosin et al. [8] in another clinical trial data set, with the several additional aims. First, we sought to assess persistence of the emergent symptoms in a data set in which the MDS-UPDRS was assessed more frequently than yearly intervals. Second, we wished to further explore the appropriate threshold for “calling” a new symptom emergent. Tosin et al. [8] used a threshold score of 1 or greater to determine symptom emergence. It is important to determine if the results look different with a threshold score of 2 or 3, as it may be difficult for study participants to distinguish between the meaning of a score of 0 and 1, or to reliably apply this difference in assessing their own level of function. Finally, we sought to explore the relationship of the ES method to conventional means of scoring the MDS-UPDRS.

## 2. Methods

Details of SURE-PD3 study have been previsouly reported. In brief, this Phase 3 study randomized 298 subjects to receive either 1000 mg inosine or matching placebo 3 times a day. The study was stopped early after an interim futility analysis revealed no difference between inosine and placebo on the rate of change in the summed score of Parts I-III of the MDS-UPDRS. Individual item-level data was extracted from the SURE-PD3 study (NCT02642393, coded as “PD-1018” in the C-path pooled dataset; download date May 21, 2021). We selected 298 participants from the SURE-PD3 study. One participant did not have any follow-up visits and this participant had no item scores evaluated at baseline. Thus, we removed this participant. We also removed unscheduled visits, telephone visits as these were most often related to adverse event occurrences and did not consistently report MDS-UPDRS data, as well as safety visits, including the washout visit.

We defined emergent symptoms (ES) as: any item with 0 score at baseline and increased to at least 1 at follow-up visits for Part IB and II items. We also assessed the ES frequency using Part IB or Part II items separately, using threshold as 1, 2 or 3.

We used item-level counts to assess the number of new symptoms that develop at each visit following baseline. Our initial definition of no symptom was a score of 0 of any Part 1B or Part 2 item. We used tables and histograms with distribution of frequencies, means, and percentages to summarize the descriptive statistics in each aim. To compare the proportion of participants with or without ES at each time point (e.g., 12 months), we used a one-sample proportion test with a null hypothesis proportion of 0.5. To test whether the proportion of ES is different in participants with initiation of symptomatic therapy (STx-yes) versus those without (STx-no), we used a two-sample proportion test. To compare the difference in number of ES among STx-yes versus STx-no, we used two-sample *t*-test and a nonparametric Wilcoxon rank-sum test. To estimate the sample size to detect a reduction in the proportion of ES by at least 30 %, we used two-sided Fisher’s exact test with a power of 0.8 and the significance level of 0.05, assuming equal group allocation.

To assess the impact of altering the ES threshold for declaring a symptom to be “emergent” we changed the definition of ES from a threshold score of 1 to a threshold score of 2 or 3 and repeated the analysis. To assess the correlation between ES and MDS-UPDRS total score, the summed score of MDS Parts I, II, and III, and the sum score of Parts Ib and II, we used both Pearson correlation and Spearman rank-order correlation coefficient. Visual and graphical methods were used to explore the stability of subject responses across visits.

To examine the stability of subjects’ responses to individual scale items in order to assess whether 1) requiring stability of change at 2 consecutive end-of-study measures would improve the validity of the method and 2) requiring two consecutive values of “0” at baseline would affect the sensitivity of the outcome, we examined the number of participants who endorsed, at consecutive visits, scores greater than or equal to 1 at baseline, week 3, week 6, week 12, and month 6, for Part IB and Part II items.

All analyses were conducted using the software R (version 4.2.3). The counts and frequencies were generated using the R package tidy-verse (version 2.0.0). The plots and figures were generated using the R package ggplot2 (version 3.4.4). The power and sample size calculation was conducted using the R package pwr (version 1.3-0).

## 3. Results

### 3.1. Baseline characteristics

The analysis dataset consisted of 297 participants and 2734 visits, with a mean follow-up of 1.55 years and SD of 0.48 years. Since there was no difference in end-of-study outcomes between the treated (Inosine) and placebo-treated groups [10], results from all participants were pooled for this analysis. Table 1 displays baseline characteristics of 297 participants.

### 3.2. Emergent symptom (ES) frequency

In general, the proportion of participants endorsing at least 1 ES at the 12-month time point was slightly less, but still comparable to the proportions reported for subjects at 12 months in the STEADY-PD study. Interestingly, 60 % of subjects endorsed at least 1 ES at the Week 3 visit when the reporting threshold was set at a score of 1. As expected, with increasing threshold to a score of 2 or 3, the number of subjects counted as reporting ES decreased accordingly. When the reporting threshold was set at 2, the proportion of subjects reporting ES increased more steadily over time (Table 2 and Supplementary Tables 1a-c)

As shown in Fig. 1a, the total number of ES reported per subject generally increased over time beginning with Month 3 of the study. However, in the early portion of the study (up to Month 6), there was actually a DECREASE in the number of ES reported when a threshold of 2 or 3 was applied. This is reflected in the pattern of MDS-UPDRS summed scores shown in Fig. 1b.

### 3.3. Stability of item responses

We next sought to examine the stability of subjects’ responses to individual scale items (Supplementary Table 2 and Supplementary Fig. 1).

**Fig. 2.**
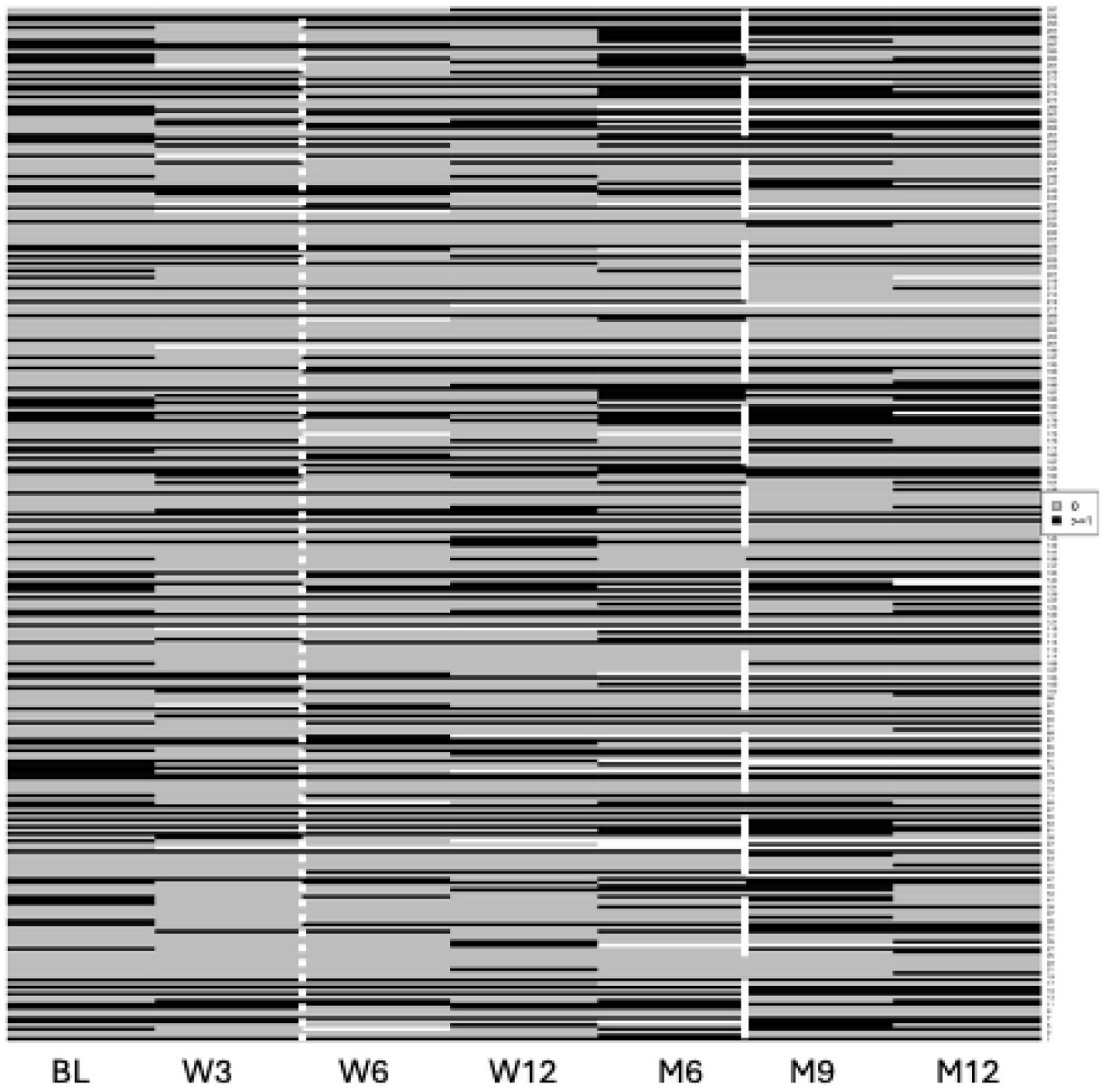
Representative heatmap plot of MDS-UPDRS item level stability and change over time Grey boxes represent item scores of 0, black represents score ≥1, and white boxes indicate missing data. Data to the left of the vertical dotted white line shows Baseline and Week 3 visits. Months 9 and 12 lie to the right of the dashed line. (Item 2.11: Getting out of bed, a car or a deep chair).

As noted above, the proportion of subjects reporting ES at the Week 3 visit was already 60 %. However, when we explored the proportion of subjects endorsing individual items with a score of 0 vs ≥ 1 at Baseline and Week 3, there was actually a DECREASE in the proportion of subjects endorsing each individual item. Fig. S1 and Table S2 illustrate the problem. For all items, fewer subjects endorsed with scores of 1 or greater at weeks 3 and 6, and the decrease in some cases lasted out to 6 months. Thus, we observed a surprising and marked instability in individual subjects’ responses, such that in some instances as many as 10 % fewer subjects endorsed an item at the 3 week visit than at the baseline visit. This resulted in the interesting observation that the mean number of items endorsed with scores ≥1 at all visits was remarkably similar (Table S2), despite the seemingly increasing proportion of subjects endorsing new symptoms at each visit. The complexity of the data is illustrated in Fig. 2, which shows the heatmap plot for MDS-UPDRS Item 2.11 (Getting out of bed, a car or a deep chair). The full set of heatmaps can be found in the online Supplement. From this one heatmap it is evident that in comparing the Month 9 and Month 12 visit, several subjects who endorsed the item at Month 12 did not do so at Month 9 and vice versa.Table S3 displays the number of items (mean and SD) with scores greater than or equal to 1 at baseline, week 3, week 6, week 12, and month 6, for Part IB and Part II items.

### 3.4. Correlation of ES with conventional MDS-UPDRS summed scores

We next explored the Pearson’s correlation between the ES events per patient and proportion of subjects reporting ES using thresholds of 1, 2 or 3 and the total summed MDS-UPDRS scores for Parts I, II and III, Ib + II, and the total for Parts I, II and III. The Pearson’s correlation coefficients for all comparisons were moderate, but most were highly statistically significant, as shown in Table 3a-c.

### 3.5. ES and symptomatic therapy

Following the example of Tosin et al. [8]) we categorized participants into two categories: STx-yes (receiving dopaminergic treatment before or at 12 months), STx-no (receiving dopaminergic treatment after 12 months or not receiving dopaminergic treatment). As shown in Table 4a-c, we compared the ES frequency and the number of ES events for those two groups of individuals. Consistent with the observations of Tosin et al. [8], there was no statistically significant difference in the number of subjects reporting ES between the STX-yes and STX-no groups at 12 months when the threshold was set at a score of 1 or 2. However, at 3 months and 6 months there was a higher proportion of subjects reporting ES in the STX-yes group than in the STX-no group (Supplementary Table S1a-c). It is tempting to speculate that the greater number of ES reported by these subjects might have contributed to the decision to initiate symptomatic treatment. Interestingly, at the end of 12 months the lack of significant difference between STX-yes and STX-no groups suggests that over longer periods of time, the ES measure is robust and resistant to the influence of medication effects. Indeed, the proportion of subjects reporting ES at the last visit prior to the introduction of symptomatic medications was higher than the proportion of subjects not receiving symptomatic medications at any point in the study. The numbers of subjects with ES when threshold was set at 3 was too small to support any conclusions. Interestingly, the number of reported ES per subject did not differ between the STx-yes and STx-no groups (Table 4d-f.

### 3.6. Effect sizes for powering clinical trials

Finally, we sought to determine if the results obtained using SURE-PD3 data would support the results of Tosin et al. [8] which were based on an analysis of the single 12-month time point in STEADY-PD3. As shown in Table 5, using the crude event rate of subjects reporting any ES with threshold greater than 1, the sample size estimates derived from the present analysis broadly support those of Tosin et al. [8]. As expected, sample size estimates would be greater of threshold scores of 2 or 3 were required to designate ES; the sample size per group would indeed more closely resemble that required for the traditional summed-score approach if the threshold for identifying ES was set at 2, for which the ES rate at 12 months as shown in Tables 2 and is 17.5 %

## 4. Discussion

In general the current investigation replicates and validates the results of Tosin et al. [8]. Using 2 time points (baseline and 12 months), a one point change and a one year interval, the frequency of ES in the SURE-PD3 study is similar to what was previously found in STEADY-PD3. At the 12-month time point, ES scores and summed MDS-UPDRS scores have a moderate correlation, but the predicted sample sizes to detect a 30 % reduction in the proportion of subjects reporting at least 1 ES across Parts Ib and II of the MDS-UPDRS are also similar, and are considerably smaller than those predicted using the combined summed score as a trial outcome measure.

The combined use of Parts I and 2 of the MDS-UPDRS has come to be recognized as a valid means of representing the impact of PD on patients’ lives, 6 10]. Part 2 itself is a valid measure of ADL function [7,11] Part 2 scores, followed by Part 1 scores, correlate most highly with the Quality of Life measures PDQ-39 and EQ-5D [11-13]. However, numerical progression using the traditional summed scoring method only registers as approximately 2 points [2,3]), and with substantial variability. This results in the need for very large sample sizes and long durations of observation if Part 2 or Parts 1b and 2 together of the MDS-UPDRS were to be used as the primary outcome measure in a clinical trial. In the absence of novel, more sensitive measures, the question arises whether alternative scoring methods could improve the sensitivity of Parts 1 and 2 of the MDS-UPDRS as a clinical trial outcome measure.

The utility of the ES measure appears to be unaffected by the introduction of symptomatic medications during the course of the trial. At 12 months there was no difference between the proportion of subjects reporting ES when the threshold score was set at 1, regardless of whether or not symptomatic treatment had been initiated during the period of observation. However, at earlier time points, the proportion of ES reporters was higher among subjects who did go on to symptomatic treatment, suggesting that ES might influence the timing of treatment initiation. When the threshold for ES reporting was set at a score of 2, the proportion of ES reporters was higher in the group that received symptomatic treatment, suggesting that this group was more rapidly progressive, and perhaps that emergent symptoms were not levodoparesponsive. The proportion of subjects reporting ES at the last visit before introduction of symptomatic medications was recorded was not less than the total proportion of subjects in the group who did not receive symptomatic medications.

In STEADY-PD3, the MDS-UPDRS was only performed at yearly intervals [4,8], whereas in SURE-PD3 it was performed at basline, 3 and 6 weeks post-baseline, then at Month 3 and every 3 months thereafter [9]. We had hoped to leverage this greater assessment frequency to assess the stability of endorsement of individual MDS-UPDRS Part Ib and II items, as the utility of ES as a clinical trial outcome measure would be greatly enhanced by requiring stable responses over at least 2 consecutive time points. However, we observed considerable fluctuation in reporting, especially early on in the study.

In the SURE study [10] there appears to have been a slight overall numerical IMPROVEMENT in reported subject function in the first 3–6 months of the study. This pattern of change in Parts I and II of the MDS-UPDRS was not in recently completed clinical trials, SPARK [14], and PASADENA [15], or ongoing observational studies (PPMI) with MDS-UPDRS performed every 2 months (SPARK [14] and PASADENA [15] OR 6 months (PPMI [3]. As can be seen from the figures in these papers average the pattern of change in MDS-UPDRS Parts I and II was by and large that of consistent and progressive worsening.

At present we cannot account for this apparent “dip” in the sum score curve. It could be explained by a number of factors: 1) the general “placebo” effect of beginning participation in a clinical trial; 2) increased familiarity of the subjects with the wording of the scale items resulting in self-perceptions of some subjects as not being as severely affected than they reported in the initial encounter; 3) sub-conscious desire of subjects to appear “worse” at the start of the study, resulting in an initial bias or 4) random, day-to-day fluctuation in mood and actual performance averaged across the study population. 5) a positive “expectation bias” on the part of the subjects, who expected to benefit from the study medication. Indeed, in a follow-on analysis, the SURE-PD3 investigators determined that the transient mean improvement in MDS-UPDRS scores could have been due to such an expectation bias on the part of the participants [16]). Thus the unusual early pattern of MDS-UPDRS scores in SURE-PD3 posed an unfortunate and insurmountable challenge to our ability to ascertain the stability of item-level scores over time.

It is widely presumed that progressive worsening of PD is tied to the anatomical spread of synuclein pathology across brain areas [17,18] affecting both dopaminergic and non-dopaminergic neuronal systems. Progression of dopamine-related dysfunction in the brain also appears to correlate with progressive loss of dopaminergic innervation in the striatum, moving from the putamen rostrally to eventually encompass the head of the caudate nucleus as seen using dopamine transporter imaging [19], and lateral-to-medial degeneration of the substantia nigra, as can be observed in vivo using neuromelanin imaging [20]. An intriguing question is whether accrual of emergent symptoms correlates with sequential involvement of new areas of the brain, which Braak’s hypothesis might predict. The present analysis did not attempt to answer this question; it is a potential area for future study. In the future, synuclein PET imaging, should a successful ligand emerge, could also be applied to explore such functional-anatomical relationships. It is also interesting to consider, that as new clinical and biological staging systems for PD and other synucleinopathies are being introduced [21,22]; the cumulative emergence of new impairments might represent an attractive stategy for basing staging classification.

It is important to note that in the present work, the instability of the measure as observed in the SURE-PD3 data set could suggest several possibilities: 1) there is actually a great deal of day-to-day fluctuation in patient’s function and self-perceived symptom severity in recently diagnosed motor/cognitive (Stage 3 PD accordijng to Simuni et al.)) [22]. Placebo effects and expectation bias might disrupt the continuity of patients’ lived experience at the beginning of a clinical trial; and 3) the SURE-PD3 dataset used as the basis of this work is in fact in some ways atypical.

Finally, we based our sample size estimations on a change from “0” to “1” in response to one or more scale items, based on evidence from development of the MDS-UPDRS scale that this interval is discernible to subjects. Therefore, an event-based analysis reliant on the number of ES using this threshold is an adequate indicator of disease activity for the purpose of Proof-of-Concept clinical trials whose purpose is to support continued development of a therapeutic agent. However, we should note that the clinical meaningfulness of a self-rated change from “0” to “1” on he various Part ib and II inventory items has not been formally established. In the course developing the MDS-UPDRS, each of the items was subjected to 2 rounds of cognitive pretesting in order to ensure the clarity of wording, the ability of patients to understand the questions, and the ability of patients to distinguish the meaning of the assigned score levels of 0–4 for each item [23]. Nonetheless, formal investigation of the clinical meaningfulness of a 0–1 step change in the response to each of the MDS-UPDRS Parts Ib and II items is warranted.

For the moment, these factors would appear to make the ES approach a suboptimal end point for pivotal clinical trials, although it may be of utility as an exploratory endpoint in Proof-of-Concept studies. Further exploration in studies in which MDS-UPDRS was administred more frequently than once per quarter, including potentially frequent remote sampling by computer or smartphone app, as has been done for the ALSFRS-R in ALS [24] is warranted. Alternate data-analytical approaches such as de-noising [25] and AUC-based methods [26] might help reduce the noisiness in the data and allow more precise estimation of the time course of appearance of new symptoms and disabilities in early motor/cognitive stages of PD.

In conclusion, we have replicated initial findings demonstrating that progressive accrual of emergent symptoms appears to be a potentially useful outcome measure for clinical trials in early-stage PD. The lack of stability of reporting individual symptoms, especially in the early months of a study may limit the utility of the ES measure in pivotal trials. However, the hypothetical reduction in the sample size required to detect a reduction in ES, compared to the large sample sizes required for traditional summed-score method of analysis [27], could potentially be advantageous for Proof-of-Concept studies. Further replication in more data-dense datasets and exploration of the patient-relevance of ES as a progression measure are warranted.

## Supplementary Material

Supplement

Supplementary data to this article can be found online at https://doi.org/10.1016/j.parkreldis.2024.107118.

## Figures and Tables

**Fig. 1a.**
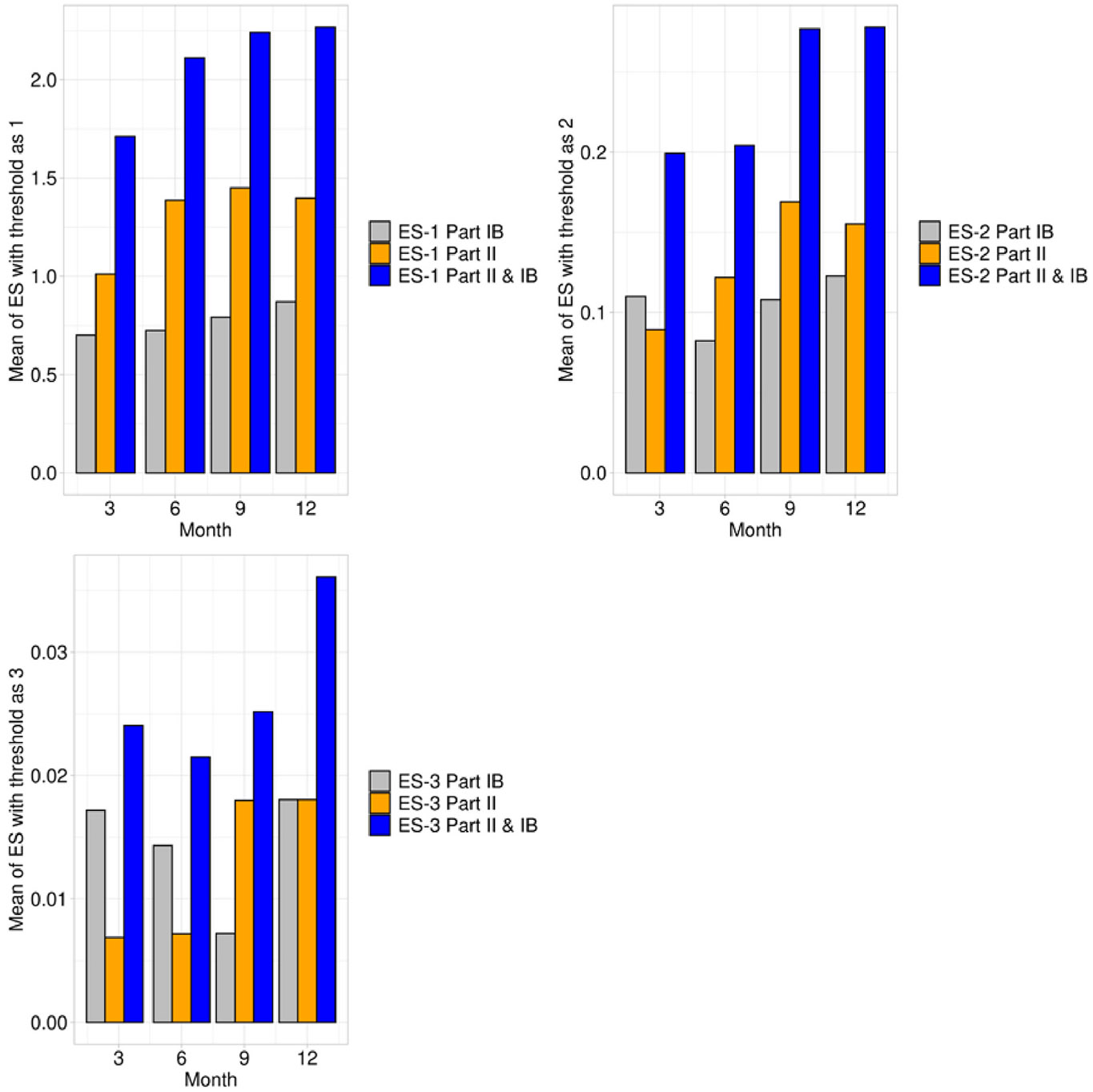
The number of ES events with threshold as 1, 2, or 3 reported at each visit.

**Fig. 1b.**
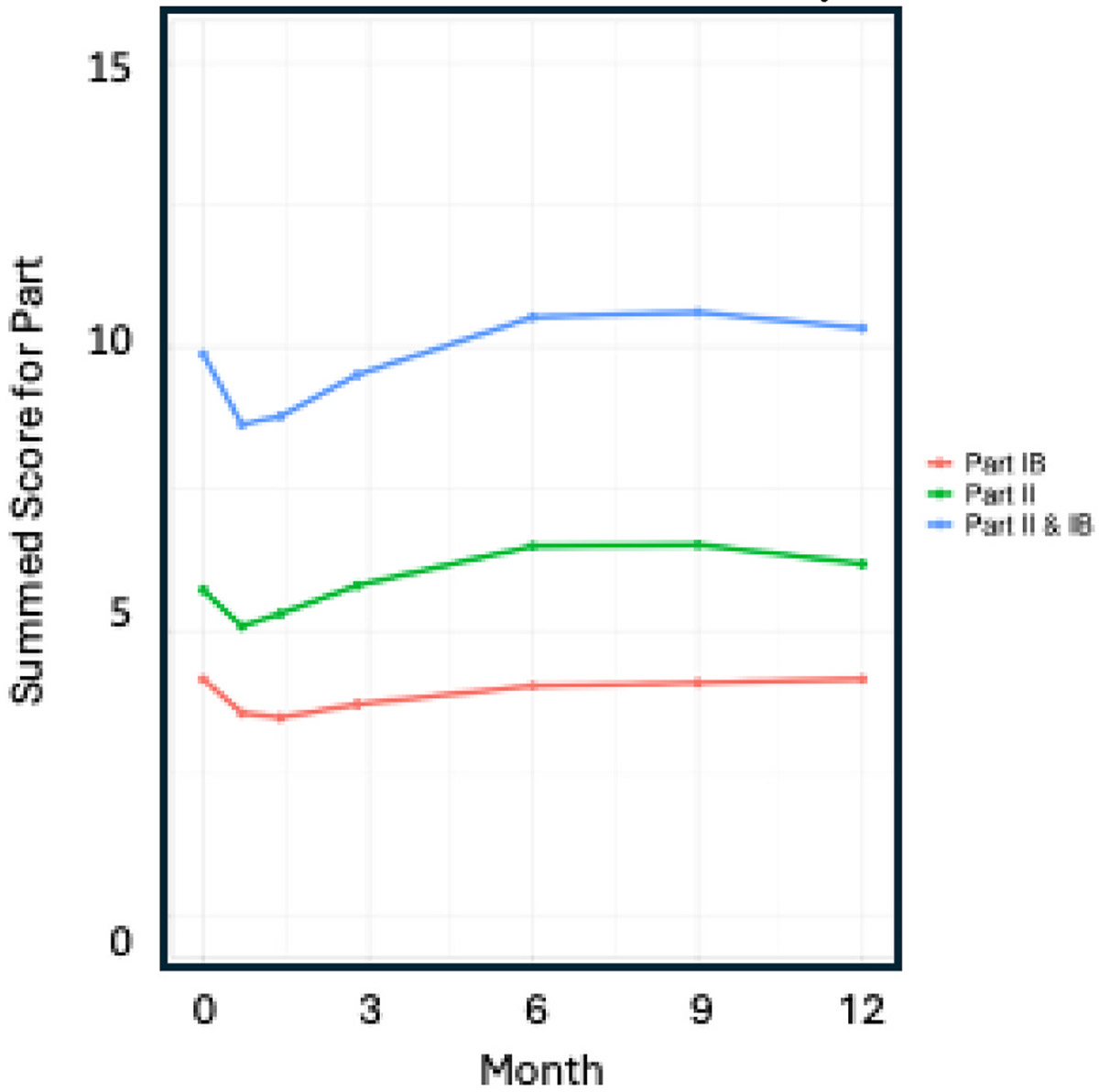
Sum of scores for Part IB and Part II by time in SURE-PD3.

**Table 1:** Baseline characteristics of 297 participants.

	297 participants
Age (years)	63.3 ± 9.6
Sex (Male)	150 (50.5 %)
Height (cm)	170.6 ± 10.5
Weight (kg)	77.6 ± 16.4
Modified H&Y Scale	1.7 ± 0.5
PD Medication	106 (35.7 %)
UPDRS-I Sum Score	5.2 ± 3.7
UPDRS-IB & II Sum Score	9.9 ± 6.3
UPDRS-II Sum Score	5.7 ± 4.2
UPDRS-III Sum Score	22 ± 8.9
Resting Tremor	240 (80.8 %)

**Table 2:** ES frequency using Part IB and II items, Part IB only, and Part II items at selected timepoints, with threshold as 1, 2 or 3.

VISIT		Part IB and II items	Part IB only	Part II items
			
	N	ES patients	ES patients	ES patients
Threshold = 1				
Week 3	290	179 (60.3 %)	112 (37.7 %)	127 (42.8 %)
Month 12	277	223 (75.1 %)	153 (51.5 %)	178 (59.9 %)
Threshold = 2				
Week 3	290	20 (6.7 %)	13 (4.4 %)	11 (3.7 %)
Month 12	277	52 (17.5 %)	28 (9.4 %)	32 (10.8 %)
Threshold = 3				
Week 3	290	4 (1.3 %)	3 (1 %)	1 (0.3 %)
Month 12	277	9 (3 %)	4 (1.3 %)	5 (1.7 %)

**Table 3a:** Pearson’s correlation and p-values between ES events per patient (top panel), number of ES patients (bottom panel), using threshold as 1, with MDS-UDPRS Part I, II, III, I + II + III, IB + II scores.

Correlation of ES events per patient and MDS-UDPRS Part I, II, III, I + II + III, IB + II scores										
	Part I	P	Part II	P	Part III	P	I + II + III	P	IB + II	P
Baseline										
Week 3	0.231	<0.001	0.342	<0.001	0.065	0.269	0.209	<0.001	0.343	<0.001
Week 6	0.186	0.001	0.304	<0.001	0.164	0.005	0.264	<0.001	0.298	<0.001
Week 12	0.322	<0.001	0.435	<0.001	0.275	<0.001	0.409	<0.001	0.450	<0.001
Month 6	0.274	<0.001	0.447	<0.001	0.254	<0.001	0.385	<0.001	0.421	<0.001
Month 9	0.339	<0.001	0.455	<0.001	0.175	0.003	0.362	<0.001	0.467	<0.001
Month 12	0.304	<0.001	0.426	<0.001	0.236	<0.001	0.382	<0.001	0.439	<0.001
										
Correlation of number of ES patients and MDS-UDPRS Part I, II, III, I + II + III, IB + II scores										
	Part I	P	Part II	P	Part III	P	I + II + III	P	IB + II	P
										
Baseline										
Week 3	0.221	<0.001	0.266	<0.001	0.033	0.581	0.158	0.007	0.285	<0.001
Week 6	0.232	<0.001	0.349	<0.001	0.254	<0.001	0.355	<0.001	0.344	<0.001
Week 12	0.282	<0.001	0.362	<0.001	0.233	<0.001	0.346	<0.001	0.379	<0.001
Month 6	0.291	<0.001	0.352	<0.001	0.191	0.001	0.318	<0.001	0.378	<0.001
Month 9	0.191	0.001	0.252	<0.001	0.140	0.020	0.226	<0.001	0.265	<0.001
Month 12	0.260	<0.001	0.289	<0.001	0.091	0.131	0.223	<0.001	0.320	<0.001

**Table 3b:** The Pearson’s correlation and p-values between ES events per patient (top panel), number of ES patients (bottom panel), with threshold as 2, and MDS-UDPRS Part I, II, III, I + II + III, IB + II scores.

Correlation of ES events per patient and MDS-UDPRS Part I, II, III, I + II + III, IB + II scores										
	Part I	P	Part II	P	Part III	P	I + II + III	P	IB + II	P
Baseline										
Week 3	0.194	<0.001	0.298	<0.001	0.212	<0.001	0.296	<0.001	0.306	<0.001
Week 6	0.168	0.004	0.164	0.005	0.106	0.071	0.175	0.003	0.208	<0.001
Week 12	0.230	<0.001	0.303	<0.001	0.109	0.065	0.228	<0.001	0.332	<0.001
Month 6	0.254	<0.001	0.342	<0.001	0.096	0.109	0.239	<0.001	0.350	<0.001
Month 9	0.347	<0.001	0.397	<0.001	−0.041	0.495	0.198	<0.001	0.429	<0.001
Month 12	0.262	<0.001	0.342	<0.001	0.116	0.055	0.255	<0.001	0.372	<0.001
										
Correlation of number of ES patients and MDS-UDPRS Part I, II, III, I + II + III, IB + II scores										
	Part I	P	Part II	P	Part III	P	I + II + III	P	IB + II	P
										
Baseline										
Week 3	0.166	0.005	0.258	<0.001	0.187	0.001	0.259	<0.001	0.269	<0.001
Week 6	0.152	0.009	0.156	0.007	0.106	0.072	0.168	0.004	0.197	<0.001
Week 12	0.230	<0.001	0.290	<0.001	0.099	0.093	0.217	<0.001	0.319	<0.001
Month 6	0.261	<0.001	0.342	<0.001	0.160	0.007	0.285	<0.001	0.338	<0.001
Month 9	0.309	<0.001	0.351	<0.001	−0.018	0.767	0.188	0.002	0.374	<0.001
Month 12	0.259	<0.001	0.319	<0.001	0.100	0.098	0.236	<0.001	0.357	<0.001

**Table 3c:** The Pearson’s correlation and p-values between ES events per patient (top panel), number of ES patients (bottom panel), with threshold as 3, and MDS-UDPRS Part I, II, III, I + II + III, IB + II scores.

Correlation of ES events per patient and MDS-UDPRS Part I, II, III, I + II + III, IB + II scores										
	Part I	P	Part II	P	Part III	P	I + II + III	P	IB + II	P
Baseline										
Week 3	0.210	<0.001	0.158	0.007	0.174	0.003	0.238	<0.001	0.210	<0.001
Week 6	0.044	0.458	−0.049	0.408	−0.035	0.552	−0.027	0.646	−0.007	0.902
Week 12	0.086	0.146	0.169	0.004	0.126	0.031	0.162	0.006	0.171	0.003
Month 6	0.174	0.004	0.156	0.009	0.112	0.061	0.172	0.004	0.189	0.002
Month 9	0.190	0.001	0.253	<0.001	0.026	0.662	0.155	0.010	0.253	<0.001
Month 12	0.186	0.002	0.052	0.390	0.079	0.190	0.123	0.041	0.137	0.022
										
Correlation of number of ES patients and MDS-UDPRS Part I, II, III, I + II + III, IB + II scores										
	Part I	P	Part II	P	Part III	P	I + II + III	P	IB + II	P
										
Baseline										
Week 3	0.210	<0.001	0.158	0.007	0.174	0.003	0.238	<0.001	0.210	<0.001
Week 6	0.044	0.458	−0.049	0.408	−0.035	0.552	−0.027	0.646	−0.007	0.902
Week 12	0.097	0.098	0.145	0.013	0.107	0.070	0.144	0.014	0.164	0.005
Month 6	0.174	0.004	0.156	0.009	0.112	0.061	0.172	0.004	0.189	0.002
Month 9	0.190	0.001	0.253	<0.001	0.026	0.662	0.155	0.010	0.253	<0.001
Month 12	0.201	<0.001	0.070	0.247	0.087	0.149	0.139	0.022	0.154	0.010

**Table 4a:** The number of subjects with ES using Part IB and II items, with threshold as 1, categorized by the use of symptomatic therapy. The p-values are based on the two-sample proportion test.

VISIT	STx-yes, N = 91 (30.6 %)		STx-no, N = 206 (69.4 %)		P-value
	
	N	ES patients	N	ES patients	
Baseline	91	0 (0 %)	206	0 (0 %)	
Week 3	90	59 (64.8 %)	200	120 (58.3 %)	0.347
Week 6	91	63 (69.2 %)	201	127 (61.7 %)	0.261
Week 12	90	73 (80.2 %)	201	137 (66.5 %)	0.024
Month 6	89	81 (89 %)	190	149 (72.3 %)	0.003
Month 9	90	74 (81.3 %)	188	152 (73.8 %)	0.209
Month 12	89	73 (80.2 %)	188	150 (72.8 %)	0.225
Last Visit Before STx	91	83 (91.2 %)			

**Table 4b:** The number of subjects with ES using Part IB and II items, with threshold as 2, categorized by the symptomatic therapy. The p-values are based on the two-sample proportion test.

VISIT	STx-yes, N = 91 (30.6 %)		STx-no, N = 206 (69.4 %)		P-value
	
	N	ES patients	N	ES patients	
Baseline	91	0 (0 %)	206	0 (0 %)	
Week 3	90	9 (9.9 %)	200	11 (5.3 %)	0.234
Week 6	91	11 (12.1 %)	201	16 (7.8 %)	0.329
Week 12	90	22 (24.2 %)	201	22 (10.7 %)	0.004
Month 6	89	15 (16.5 %)	190	27 (13.1 %)	0.556
Month 9	90	27 (29.7 %)	188	29 (14.1 %)	0.003
Month 12	89	22 (24.2 %)	188	30 (14.6 %)	0.065
Last Visit Before STx	91	28 (30.8 %)			

**Table 4c:** The number of subjects with ES using Part IB and II items, with threshold as 3, categorized by the symptomatic therapy. The p-values are based on the two-sample proportion test.

VISIT	STx-yes, N = 91 (30.6 %)		STx-no, N = 206 (69.4 %)		P-value
	
	N	ES patients	N	ES patients	
Baseline	91	0 (0 %)	206	0 (0 %)	
Week 3	90	1 (1.1 %)	200	3 (1.5 %)	>0.90
Week 6	91	0 (0 %)	201	3 (1.5 %)	0.598
Week 12	90	2 (2.2 %)	201	4 (1.9 %)	>0.90
Month 6	89	3 (3.3 %)	190	3 (1.5 %)	0.554
Month 9	90	3 (3.3 %)	188	4 (1.9 %)	0.768
Month 12	89	4 (4.4 %)	188	5 (2.4 %)	0.586
Last Visit Before STx	91	5 (5.5 %)			

**Table 4d:** The number of ES events per subject, using Part IB and II items, with threshold as 1, categorized by the symptomatic therapy.

VISIT	STx-yes, N = 91 (30.6 %)		STx-no, N = 206 (69.4 %)		P-value (T-test)	P-value (Wilcoxon)
	
	N	Mean (SD)	N	Mean (SD)		
Baseline	91	0 (0)	206	0 (0)		
Week 3	90	1.36 (1.60)	200	1.15 (1.27)	0.285	0.443
Week 6	91	1.41 (1.42)	201	1.22 (1.37)	0.305	0.236
Week 12	90	1.97 (1.75)	201	1.60 (1.63)	0.091	0.065
Month 6	89	2.30 (1.92)	190	2.02 (1.91)	0.254	0.156
Month 9	90	2.38 (2.25)	188	2.18 (1.79)	0.456	0.978
Month 12	89	2.10 (2.03)	188	2.35 (2.06)	0.353	0.262
Last Visit Before STx	91	2.74 (2.07)				

**Table 4e:** The number of ES events ES events per subject, using Part IB and II items, with threshold as 2, categorized by the symptomatic therapy.

VISIT	STx-yes, N = 91 (30.6 %)		STx-no, N = 206 (69.4 %)		P-value (T-test)	P-value (Wilcoxon)
	
	N	Mean (SD)	N	Mean (SD)		
Baseline	91	0 (0)	206	0 (0)	NA	NA
Week 3	90	0.14 (0.49)	200	0.07 (0.33)	0.188	0.156
Week 6	91	0.13 (0.37)	201	0.09 (0.32)	0.348	0.265
Week 12	90	0.31 (0.70)	201	0.15 (0.48)	0.047	0.004
Month 6	89	0.26 (0.72)	190	0.18 (0.49)	0.345	0.519
Month 9	90	0.42 (0.85)	188	0.21 (0.56)	0.031	0.005
Month 12	89	0.29 (0.55)	188	0.27 (0.71)	0.788	0.145
Last Visit Before STx	91	0.44 (0.82)				

**Table 4f:** The number ES events per subject, using Part IB and II items, with threshold as 3, categorized by the symptomatic therapy.

VISIT	STx-yes, N = 91 (30.6 %)		STx-no, N = 206 (69.4 %)		P-value (T-test)	P-value (Wilcoxon)
	
	N	Mean (SD)	N	Mean (SD)		
Baseline	91	0 (0)	206	0 (0)	NA	NA
Week 3	90	0.01 (0.11)	200	0.01 (0.12)	0.782	0.796
Week 6	91	0 (0)	201	0.01 (0.12)	0.083	0.244
Week 12	90	0.03 (0.23)	201	0.02 (0.14)	0.615	0.890
Month 6	89	0.03 (0.18)	190	0.02 (0.12)	0.401	0.339
Month 9	90	0.03 (0.18)	188	0.02 (0.14)	0.580	0.551
Month 12	89	0.06 (0.28)	188	0.03 (0.16)	0.351	0.416
Last Visit Before STx	91	0.07 (0.29)				

**Table 5:** Sample size estimates under various scenarios to detect 30 % reduction in event rate, based on type I error of 0.05, and power of 80 %.

Effect Size	No drop-out	Drop-out rate 10 %	Drop-out rate 15 %	Drop-out rate 20 %
Event rate change from 75.1 % to 52.6 %	N = 78	N = 87 per	N = 92 per	N = 98 per
	per arm	arm	arm	arm
	N = 156	N = 174	N = 184	N = 196
	total	total	total	total

Note: The sample size calculation is based on a two-sided, two-sample Fisher’s Exact test.
