# Supplementary material for: Validating new symptom emergence as a patient-centric outcome measure for PD clinical trials
Source: Parkinsonism Relat Disord. Author manuscript; Available in PMC 2025 Sep 9. (PMC12418693; doi:10.1016/j.parkreldis.2024.107118)

**Validating New Symptom Emergence as a Patient-Centric Outcome Measure for PD Clinical Trials**

**Supplementary Data**

**Table S1a. ES frequency using Part IB and II items, Part IB only, and Part II items, with threshold as 1.**

|  |  | **Part IB and II items** | **Part IB only** | **Part II items** |
| --- | --- | --- | --- | --- |
| **VISIT** | N | ES patients | ES patients | ES patients |
| **Baseline** | 297 | 0 (0%) | 0 (0%) | 0 (0%) |
| **Week 3** | 290 | 179 (60.3%) | 112 (37.7%) | 127 (42.8%) |
| **Week 6** | 293 | 191 (64.3%) | 114 (38.4%) | 133 (44.8%) |
| **Week 12** | 291 | 210 (70.7%) | 137 (46.1%) | 159 (53.5%) |
| **Month 6** | 279 | 230 (77.4%) | 141 (47.5%) | 186 (62.6%) |
| **Month 9** | 278 | 226 (76.1%) | 145 (48.8%) | 183 (61.6%) |
| **Month 12** | 277 | 223 (75.1%) | 153 (51.5%) | 178 (59.9%) |

**Table S1b. ES frequency using Part IB and II items, Part IB only, and Part II items, with threshold as 2**.

|  |  | **Part IB and II items** | **Part IB only** | **Part II items** |
| --- | --- | --- | --- | --- |
| **VISIT** | N | ES patients | ES patients | ES patients |
| **Baseline** | 297 | 0 (0%) | 0 (0%) | 0 (0%) |
| **Week 3** | 290 | 20 (6.7%) | 13 (4.4%) | 11 (3.7%) |
| **Week 6** | 293 | 27 (9.1%) | 15 (5.1%) | 13 (4.4%) |
| **Week 12** | 291 | 44 (14.8%) | 29 (9.8%) | 21 (7.1%) |
| **Month 6** | 279 | 42 (14.1%) | 20 (6.7%) | 28 (9.4%) |
| **Month 9** | 278 | 56 (18.9%) | 26 (8.8%) | 38 (12.8%) |
| **Month 12** | 277 | 52 (17.5%) | 28 (9.4%) | 32 (10.8%) |

**Table S1c. ES frequency using Part IB and II items, Part IB only, and Part II items, with threshold as 3.**

|  |  | **Part IB and II items** | **Part IB only** | **Part II items** |
| --- | --- | --- | --- | --- |
| **VISIT** | N | ES patients | ES patients | ES patients |
| **Baseline** | 297 | 0 (0%) | 0 (0%) | 0 (0%) |
| **Week 3** | 290 | 4 (1.3%) | 3 (1%) | 1 (0.3%) |
| **Week 6** | 293 | 3 (1%) | 2 (0.7%) | 1 (0.3%) |
| **Week 12** | 291 | 6 (2%) | 5 (1.7%) | 2 (0.7%) |
| **Month 6** | 279 | 6 (2%) | 4 (1.3%) | 2 (0.7%) |
| **Month 9** | 278 | 7 (2.4%) | 2 (0.7%) | 5 (1.7%) |
| **Month 12** | 277 | 9 (3%) | 4 (1.3%) | 5 (1.7%) |

**Table S2. Number and percent of participants endorsing scores greater than or equal to 1 at baseline, week 3, week 6, week 12, and month 6, for Part IB and Part II individual items and total Part IB and Part II scores.**

|  | Baseline  (N = 297) | | Week 3  (N = 290) | | Week 6  (N = 293) | | Week 12  (N = 291) | | Month 6  (N = 279) | |
| --- | --- | --- | --- | --- | --- | --- | --- | --- | --- | --- |
| Item 1.7 | 167 | 56% | 130 | 45% | 132 | 45% | 136 | 47% | 124 | 44% |
| Item 1.8 | 167 | 56% | 135 | 47% | 130 | 44% | 132 | 45% | 146 | 52% |
| Item 1.9 | 176 | 59% | 156 | 54% | 155 | 53% | 166 | 57% | 159 | 57% |
| Item 1.10 | 131 | 44% | 121 | 42% | 127 | 43% | 133 | 46% | 118 | 42% |
| Item 1.11 | 83 | 28% | 73 | 25% | 72 | 25% | 70 | 24% | 78 | 28% |
| Item 1.12 | 73 | 25% | 61 | 21% | 56 | 19% | 62 | 21% | 57 | 20% |
| Item 1.13 | 163 | 55% | 131 | 45% | 132 | 45% | 137 | 47% | 147 | 53% |
| Part IB Total | 271 | 91% | 252 | 87% | 249 | 85% | 253 | 87% | 252 | 90% |
| Item 2.1 | 89 | 30% | 74 | 26% | 69 | 24% | 89 | 31% | 87 | 31% |
| Item 2.2 | 103 | 35% | 96 | 33% | 100 | 34% | 109 | 37% | 104 | 37% |
| Item 2.3 | 41 | 14% | 24 | 8% | 23 | 8% | 33 | 11% | 36 | 13% |
| Item 2.4 | 96 | 32% | 81 | 28% | 87 | 30% | 104 | 36% | 103 | 37% |
| Item 2.5 | 115 | 39% | 100 | 34% | 118 | 40% | 121 | 42% | 123 | 44% |
| Item 2.6 | 76 | 26% | 62 | 21% | 66 | 23% | 79 | 27% | 83 | 30% |
| Item 2.7 | 176 | 59% | 156 | 54% | 167 | 57% | 175 | 60% | 173 | 62% |
| Item 2.8 | 97 | 33% | 86 | 30% | 86 | 29% | 102 | 35% | 126 | 45% |
| Item 2.9 | 72 | 24% | 72 | 25% | 72 | 25% | 75 | 26% | 82 | 29% |
| Item 2.10 | 274 | 92% | 261 | 90% | 267 | 91% | 264 | 91% | 254 | 91% |
| Item 2.11 | 108 | 36% | 83 | 29% | 91 | 31% | 98 | 34% | 117 | 42% |
| Item 2.12 | 112 | 38% | 96 | 33% | 95 | 32% | 94 | 32% | 112 | 40% |
| Item 2.13 | 16 | 5% | 14 | 5% | 13 | 4% | 12 | 4% | 21 | 8% |
| Part II Total | 296 | 100% | 287 | 99% | 287 | 98% | 288 | 99% | 275 | 99% |

**Table S3a. Mean (SD) of items per subject with scores greater than or equal to 1 at baseline, week 3, week 6, week 12, and month 6, for Part IB and Part II.**

|  | Baseline | Week 3 | Week 6 | Week 12 | Month 6 |
| --- | --- | --- | --- | --- | --- |
| N | 297 | 290 | 293 | 291 | 279 |
| Part 1B | 3.23 (1.87) | 2.78 (1.83) | 2.74 (1.89) | 2.87 (1.93) | 2.97 (1.88) |
| Part II | 4.63 (3.00) | 4.16 (2.89) | 4.28 (2.94) | 4.66 (3.15) | 5.09 (3.24) |
| Part 1B and II | 7.86 (4.24) | 6.94 (4.14) | 7.02 (4.23) | 7.53 (4.45) | 8.06 (4.56) |

**Table S3b. Mean (SD) of sum of scores at baseline, week 3, week 6, week 12, and month 6, for Part IB and Part II.**

|  | Baseline | Week 3 | Week 6 | Week 12 | Month 6 |
| --- | --- | --- | --- | --- | --- |
| N | 297 | 290 | 293 | 291 | 279 |
| Part 1B | 4.15 (2.94) | 3.56 (2.82) | 3.47 (2.95) | 3.71 (2.97) | 4.03 (3.14) |
| Part II | 5.72 (4.18) | 5.08 (3.87) | 5.31 (4.14) | 5.80 (4.37) | 6.49 (4.62) |
| Part 1B & II | 9.87 (6.25) | 8.63 (5.98) | 8.78 (6.34) | 9.51 (6.50) | 10.53 (6.92) |

**Figure S1a. Proportion of participants with scores greater than or equal to 1 at baseline, week 3, week 6, week 12, and month 6, for individual Part IB items.**


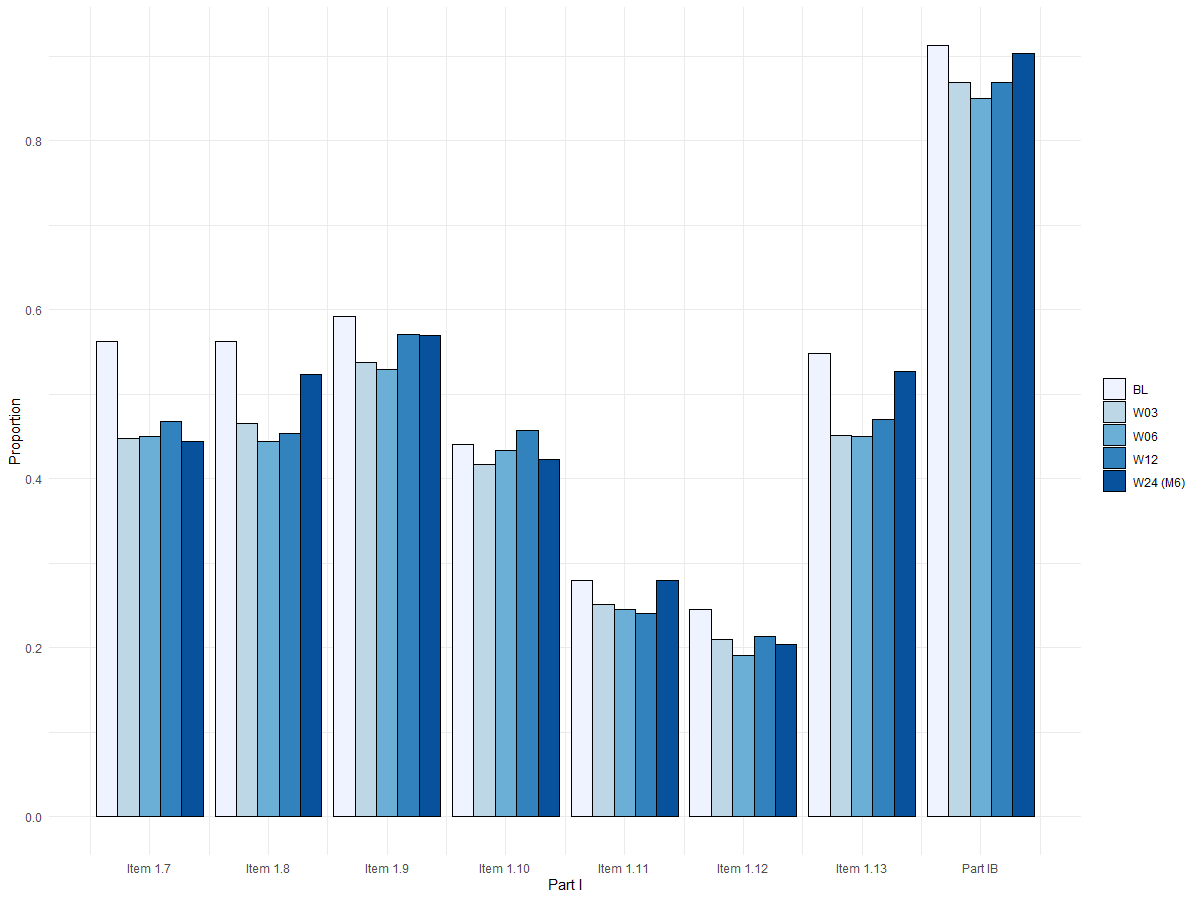


**Figure S1b. Proportion of participants with scores greater than or equal to 1 at baseline, week 3, week 6, week 12, and month 6, for individual Part II items.**


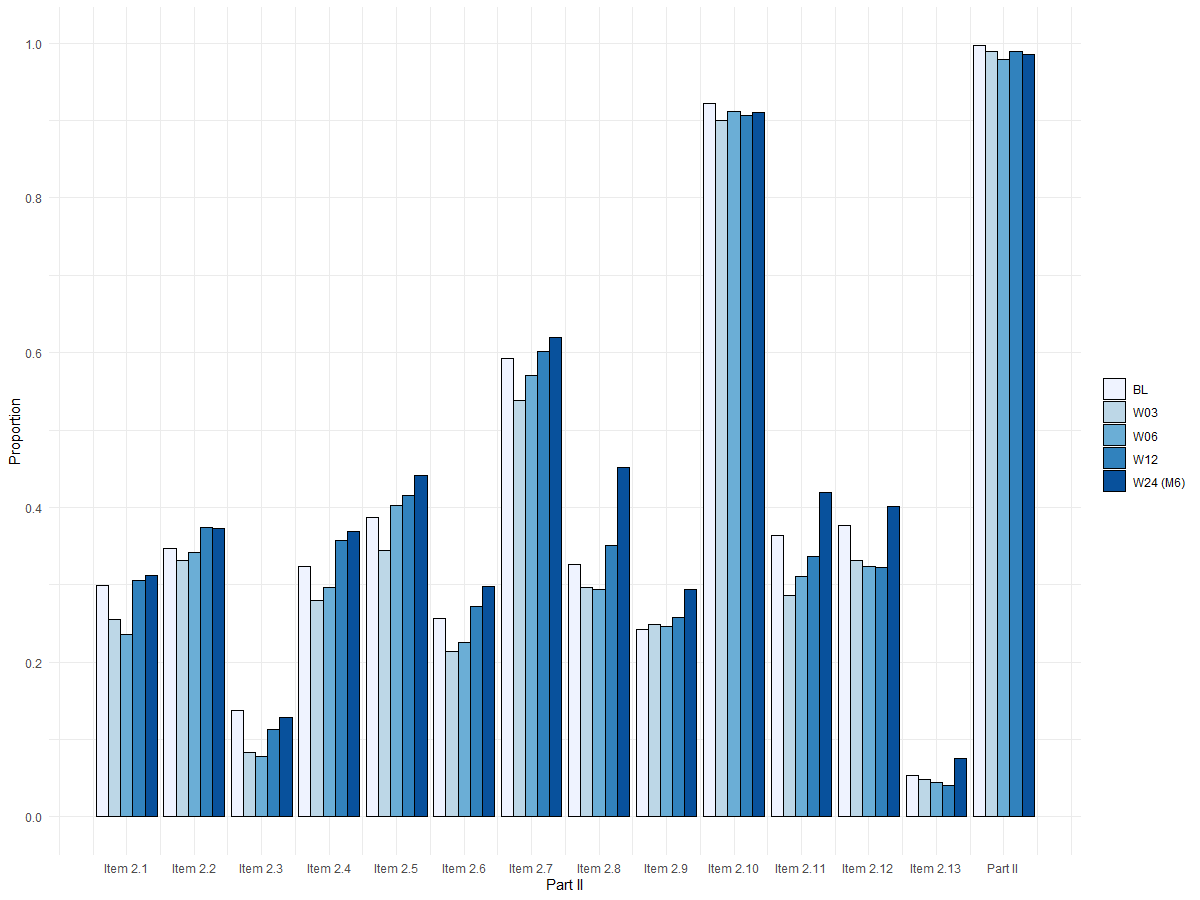


**Figure S2 Heatmaps of Individual MDS-UPDRS Part Ib and 2 item scores in SURE-PD3**


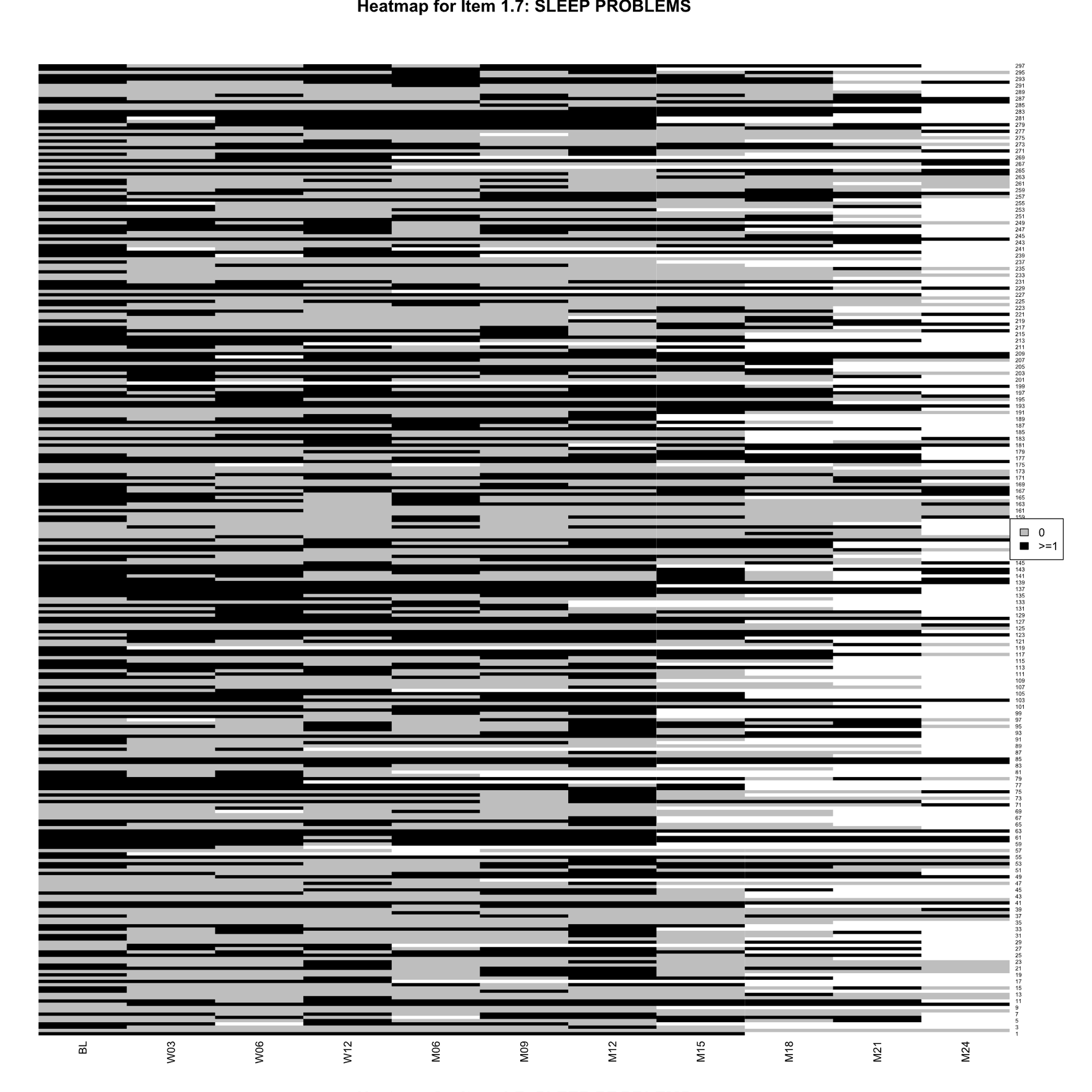

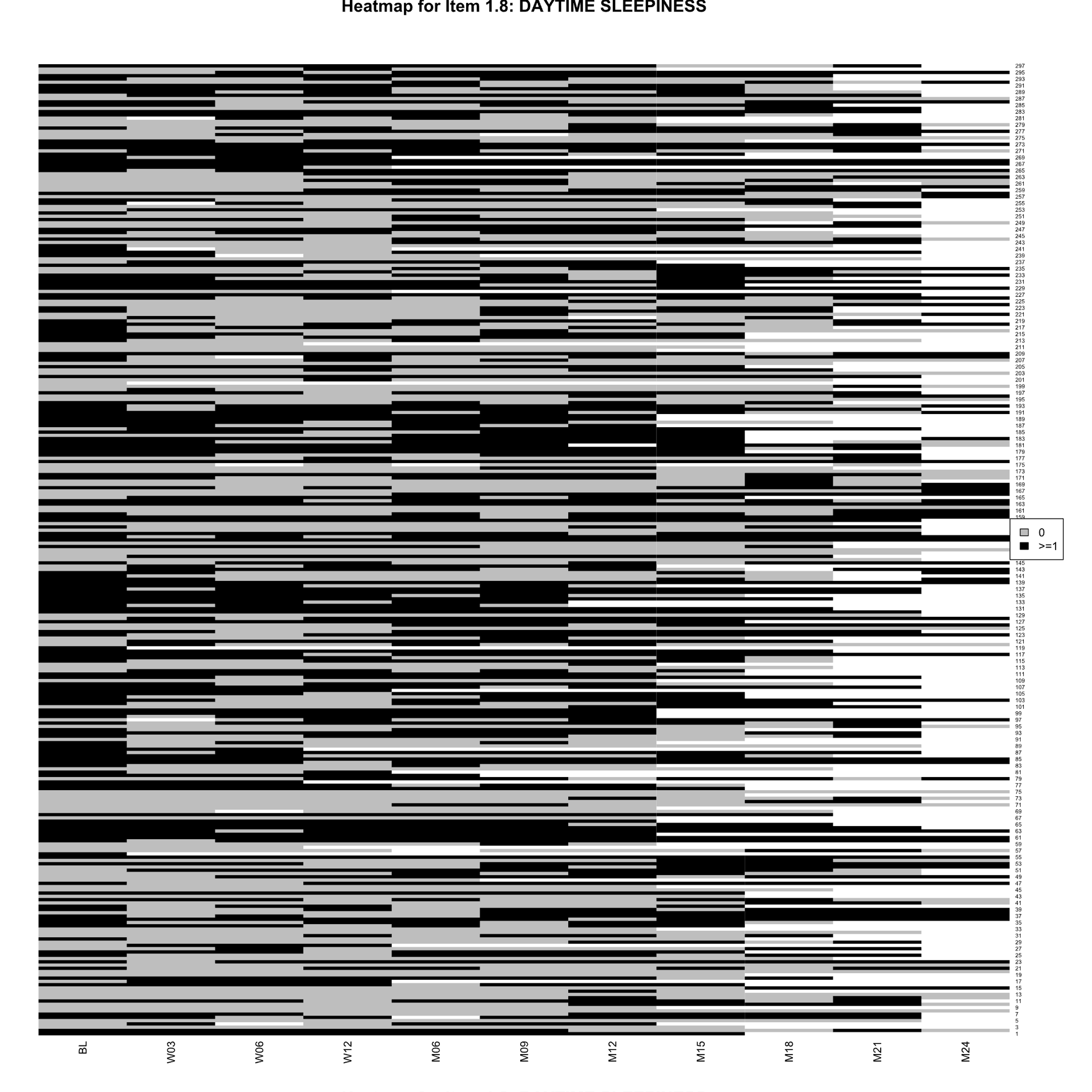

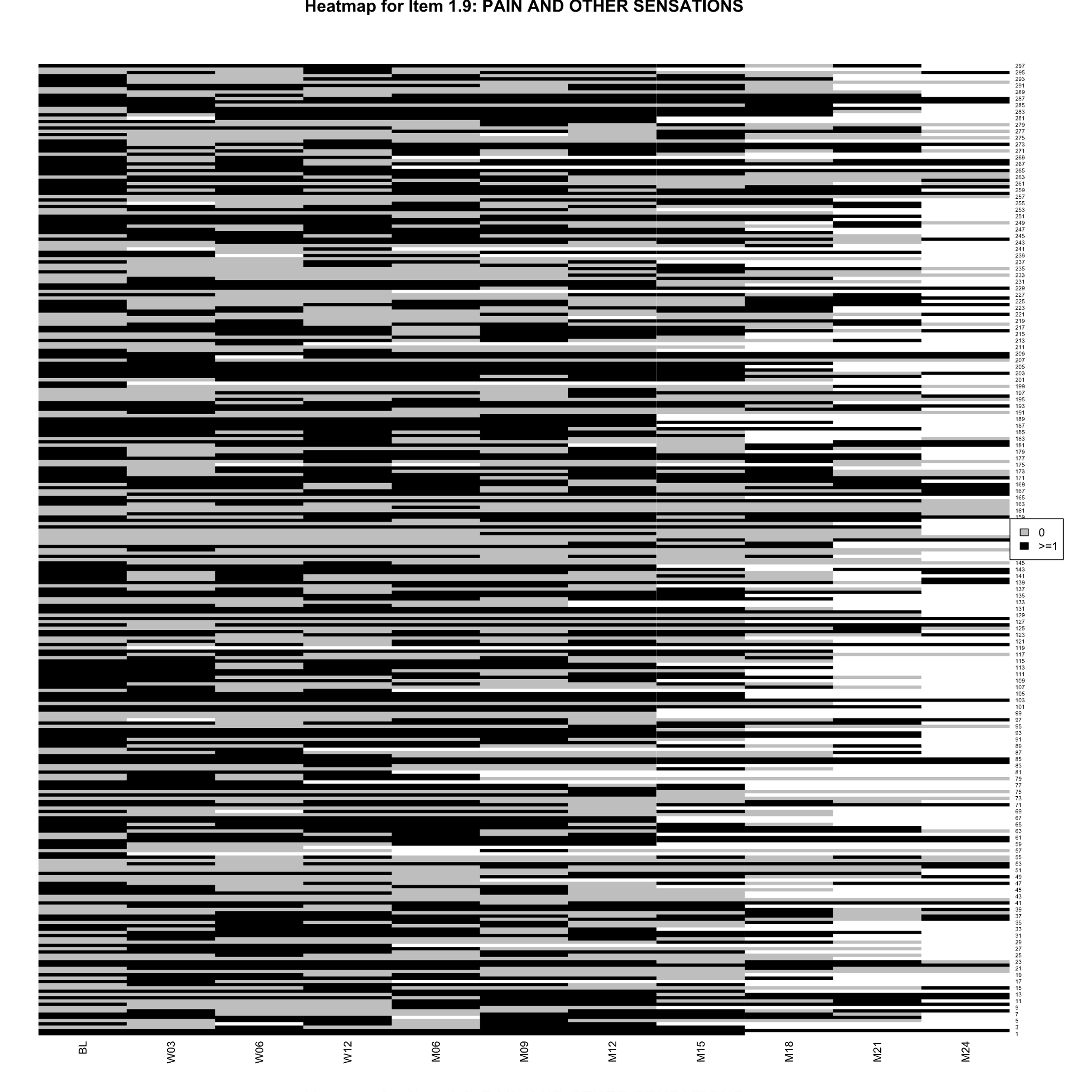

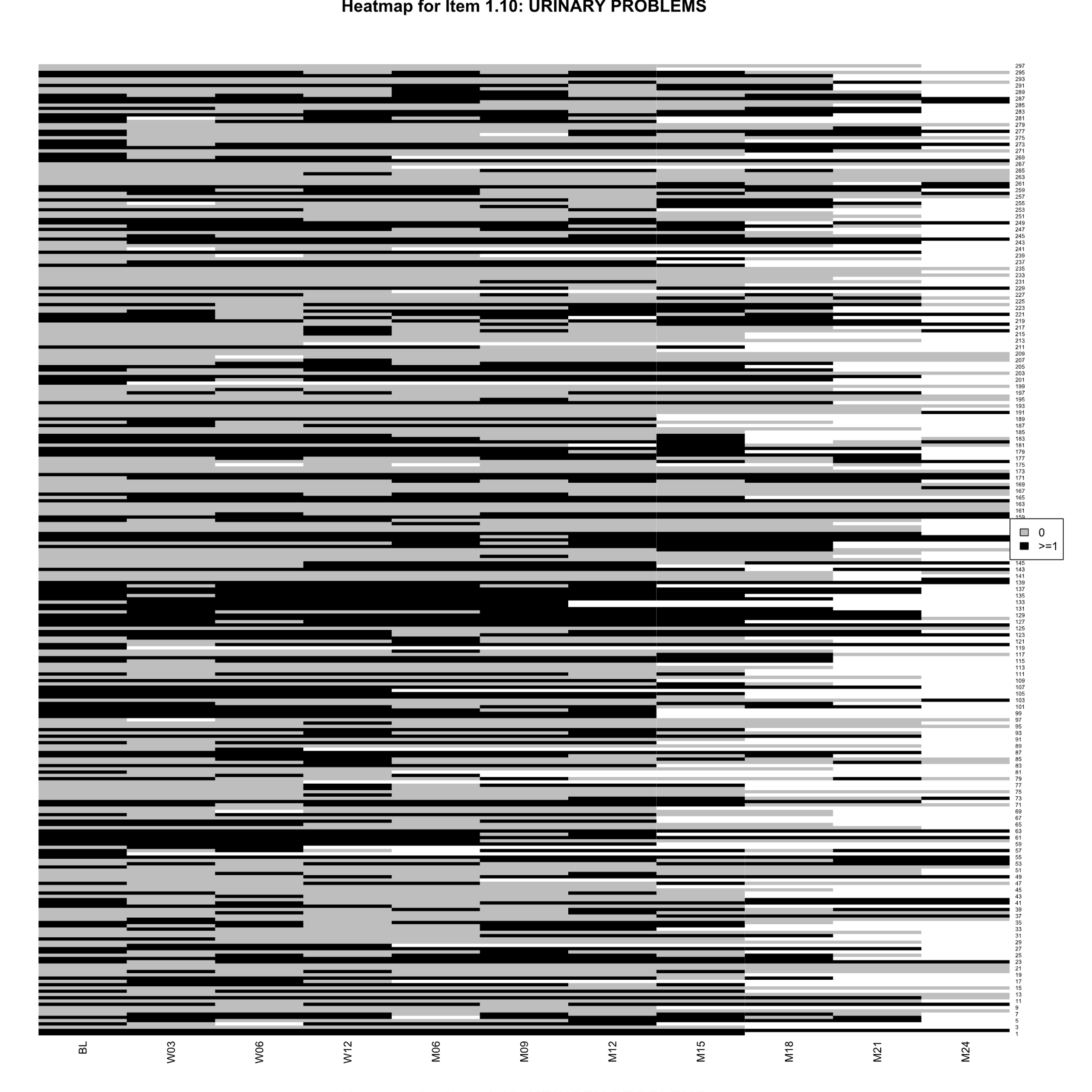

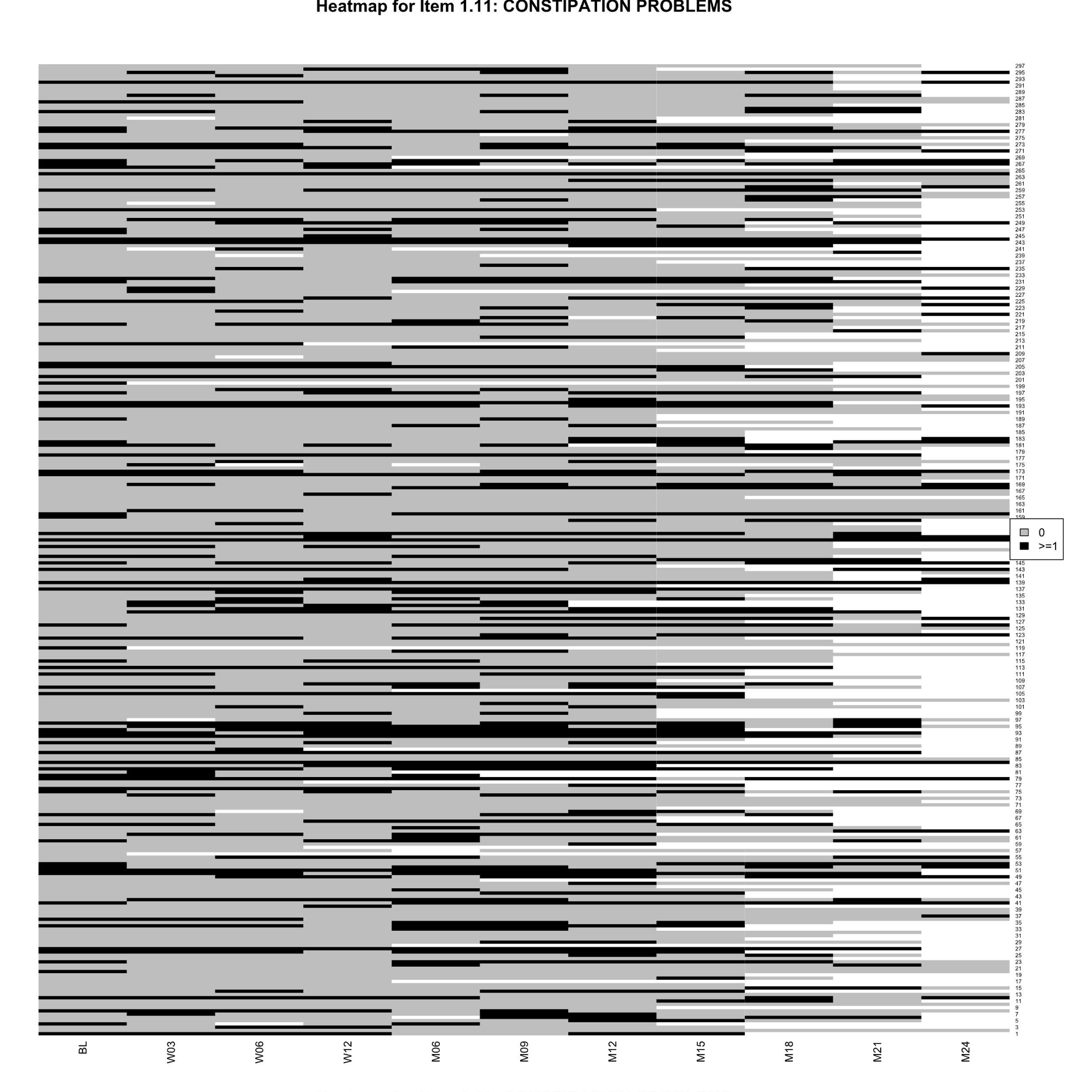

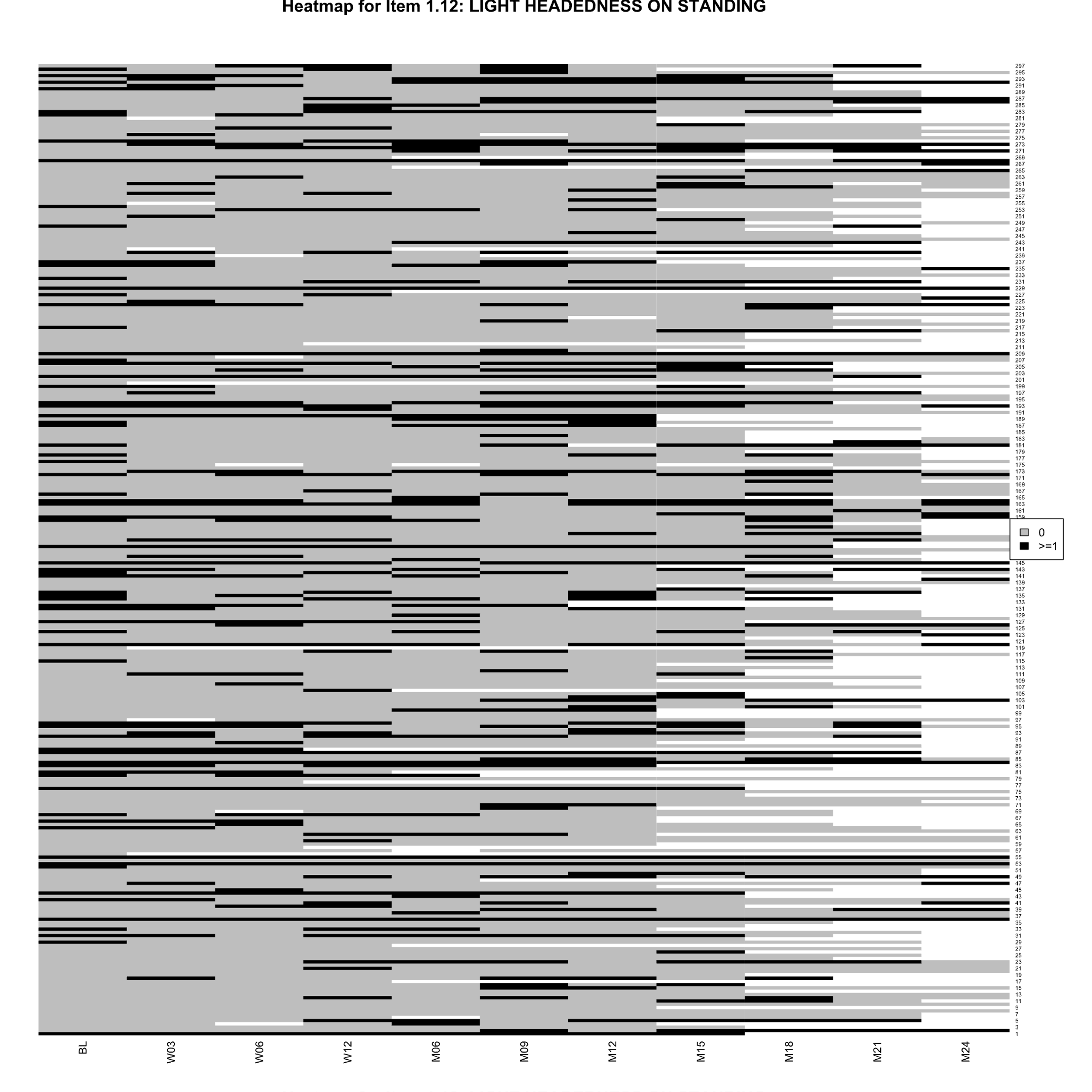

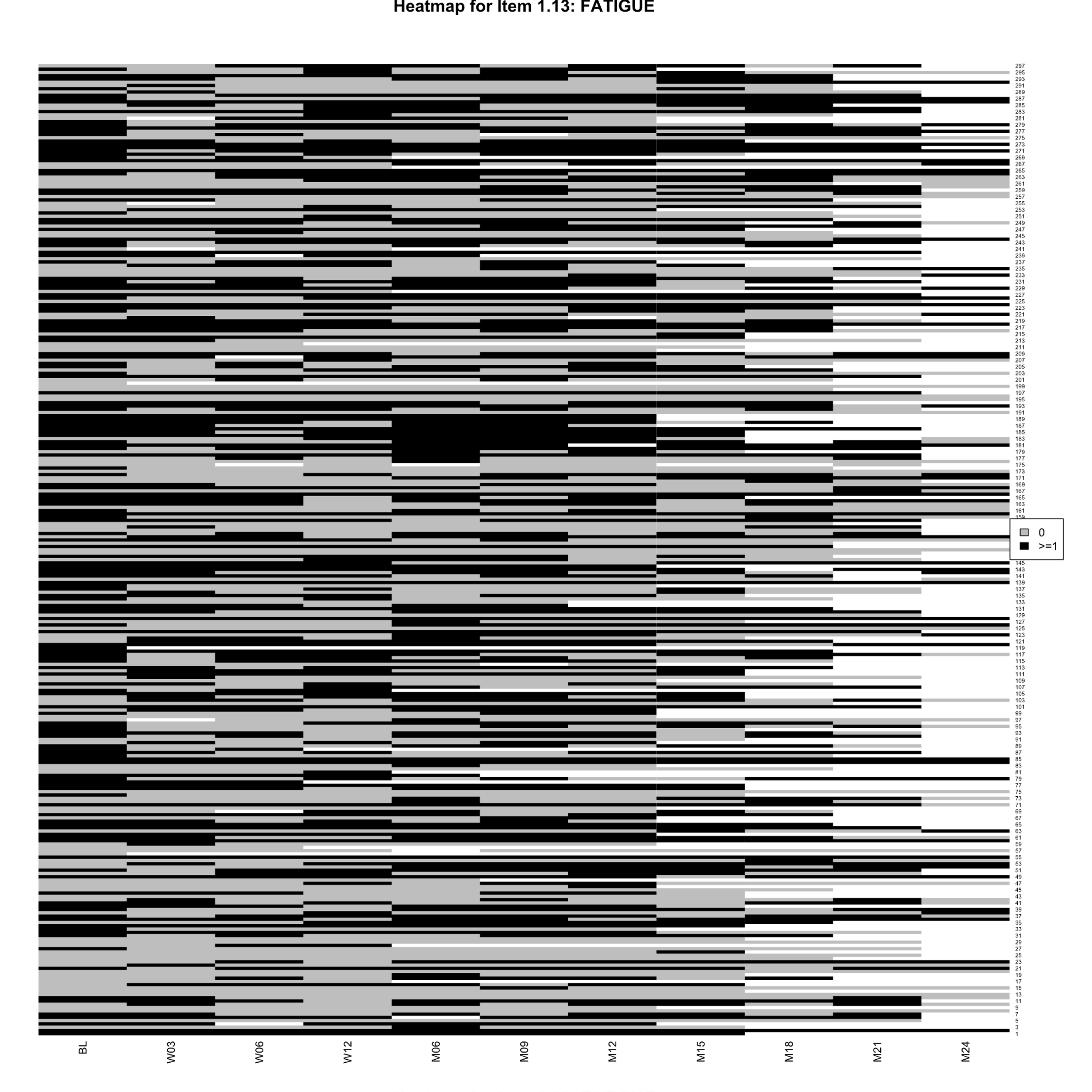

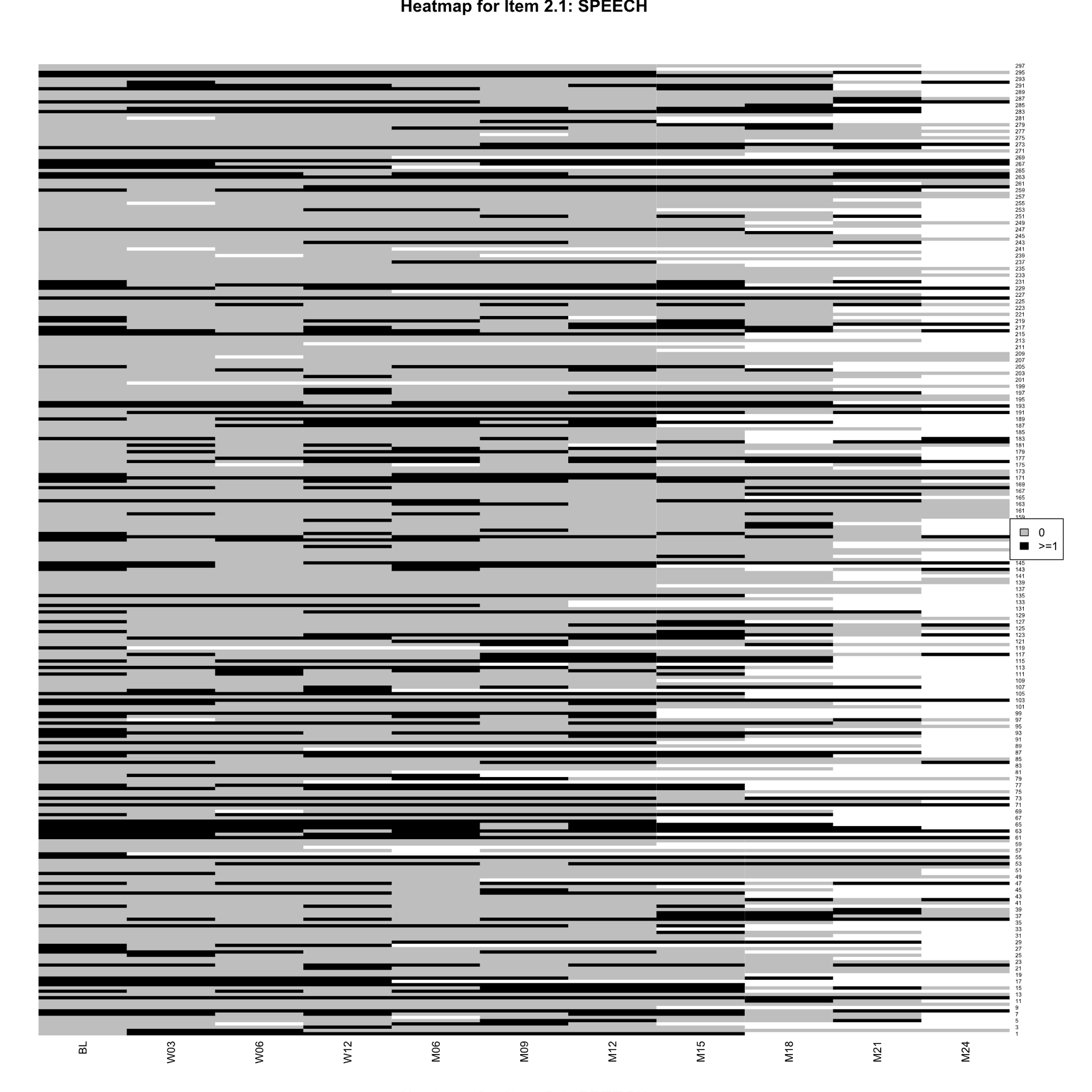

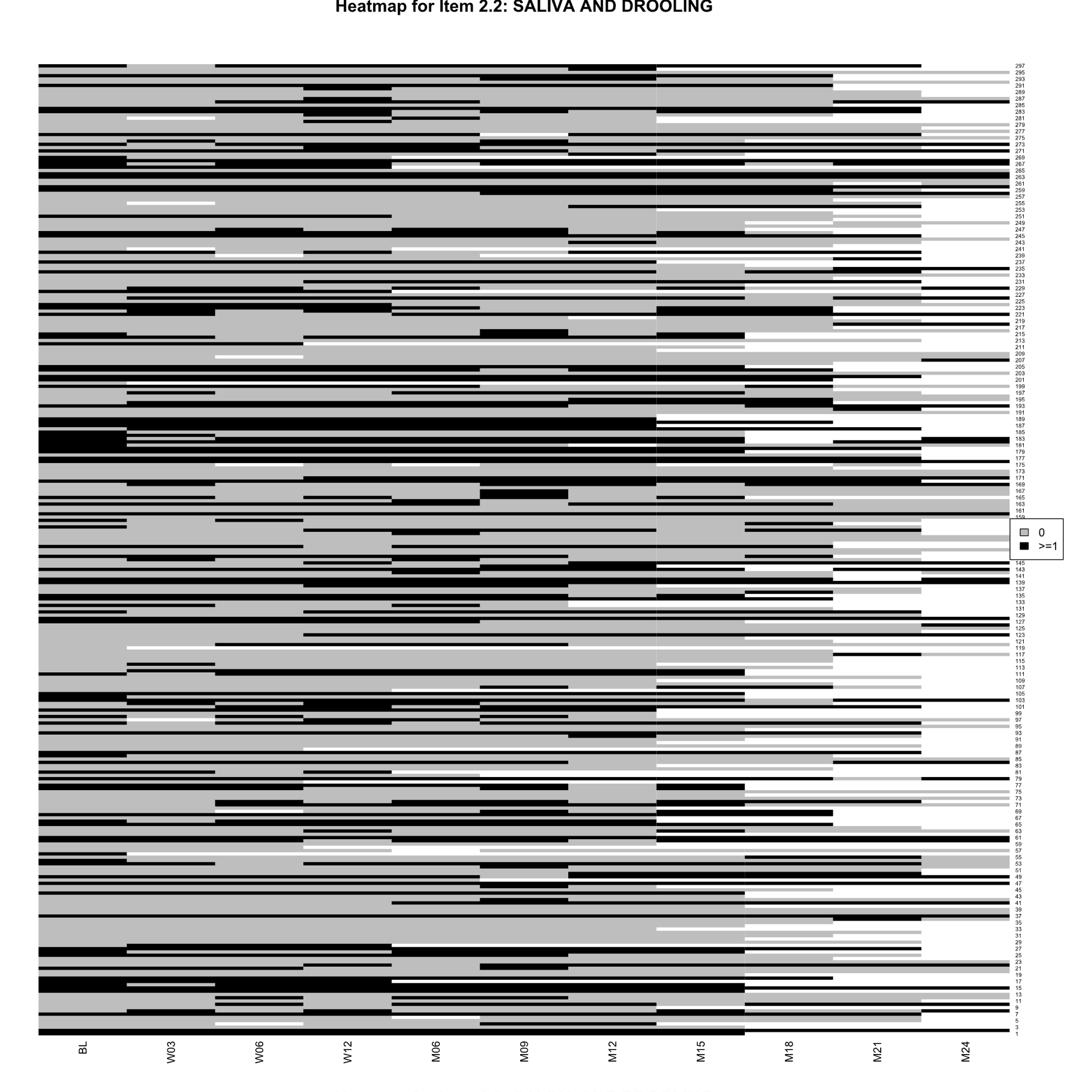

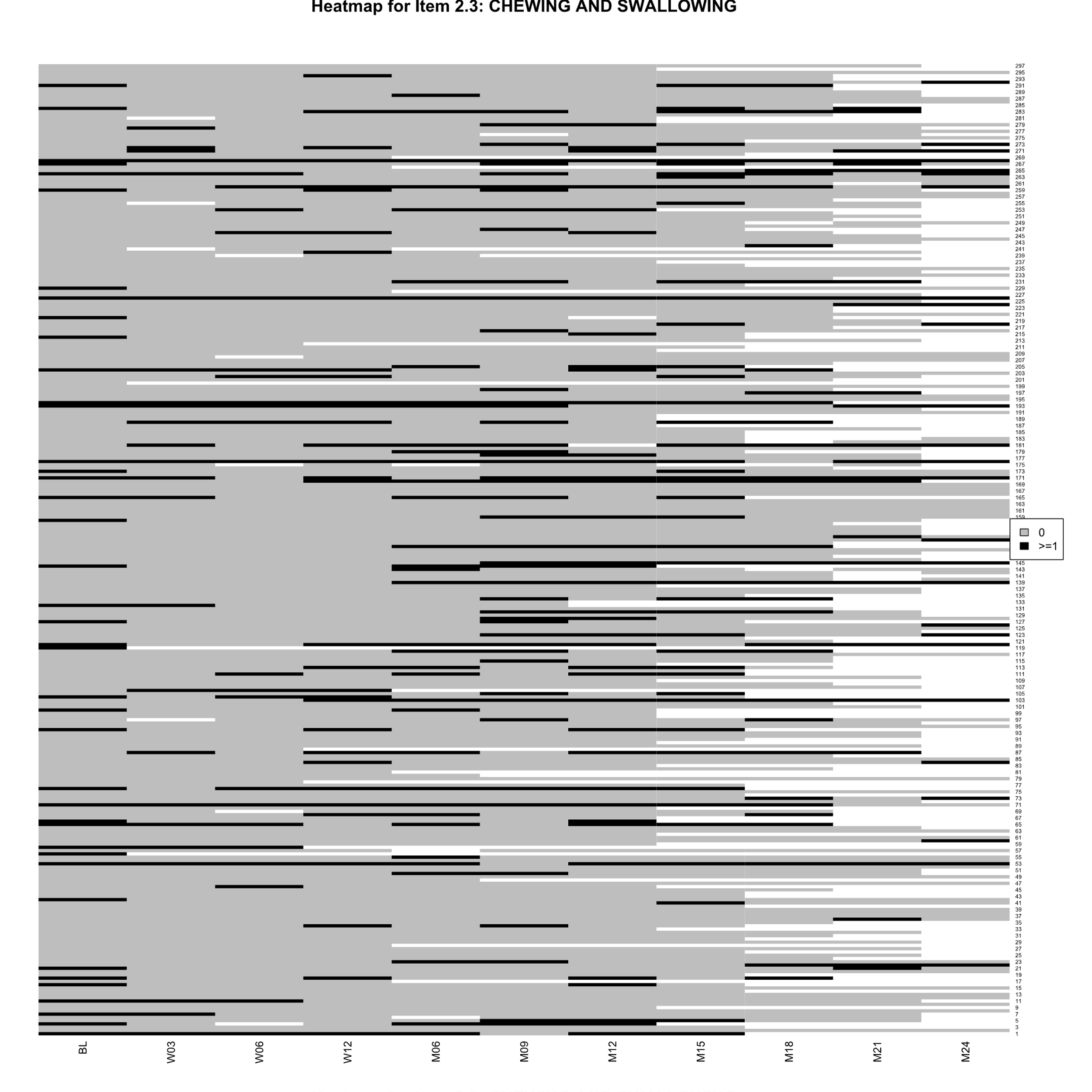

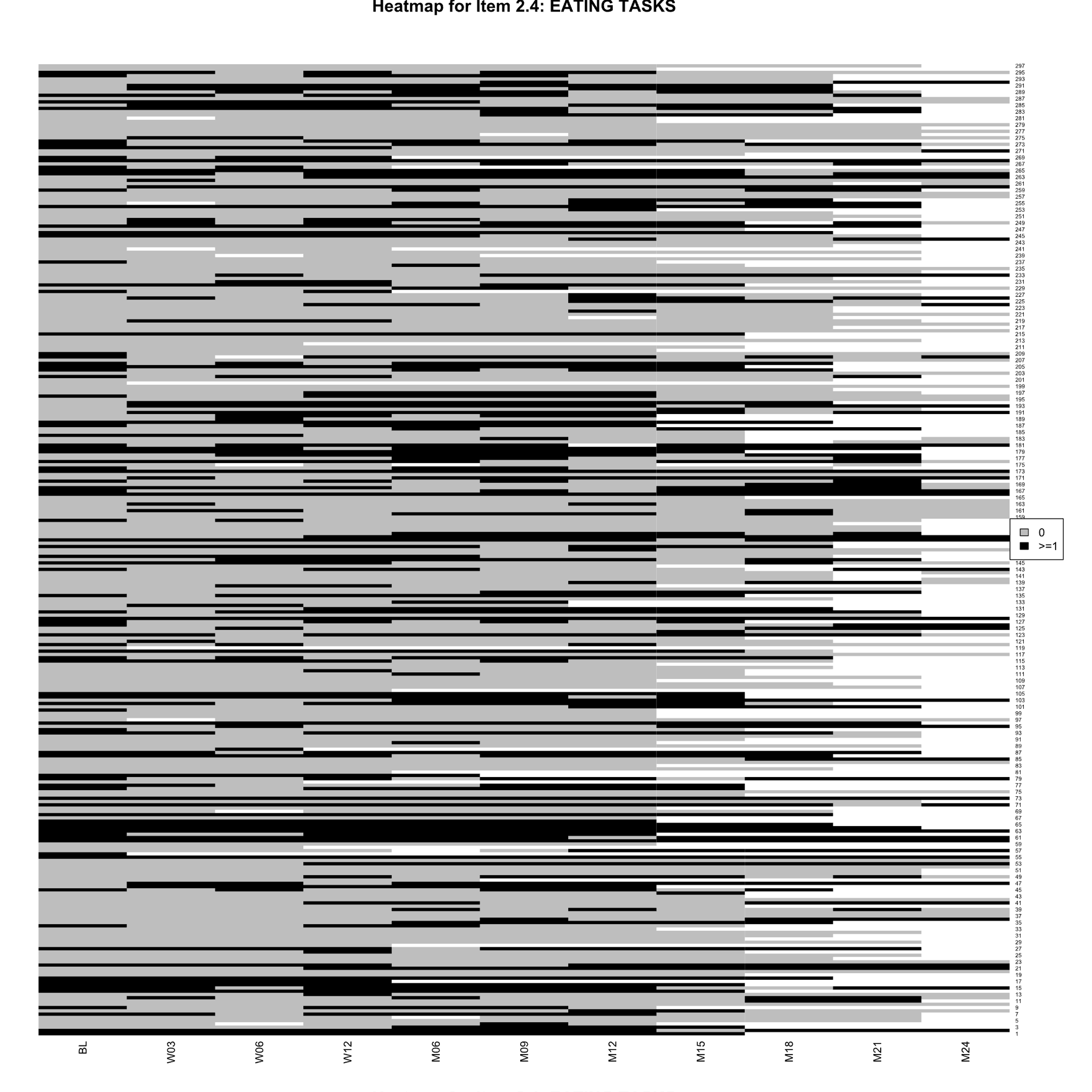

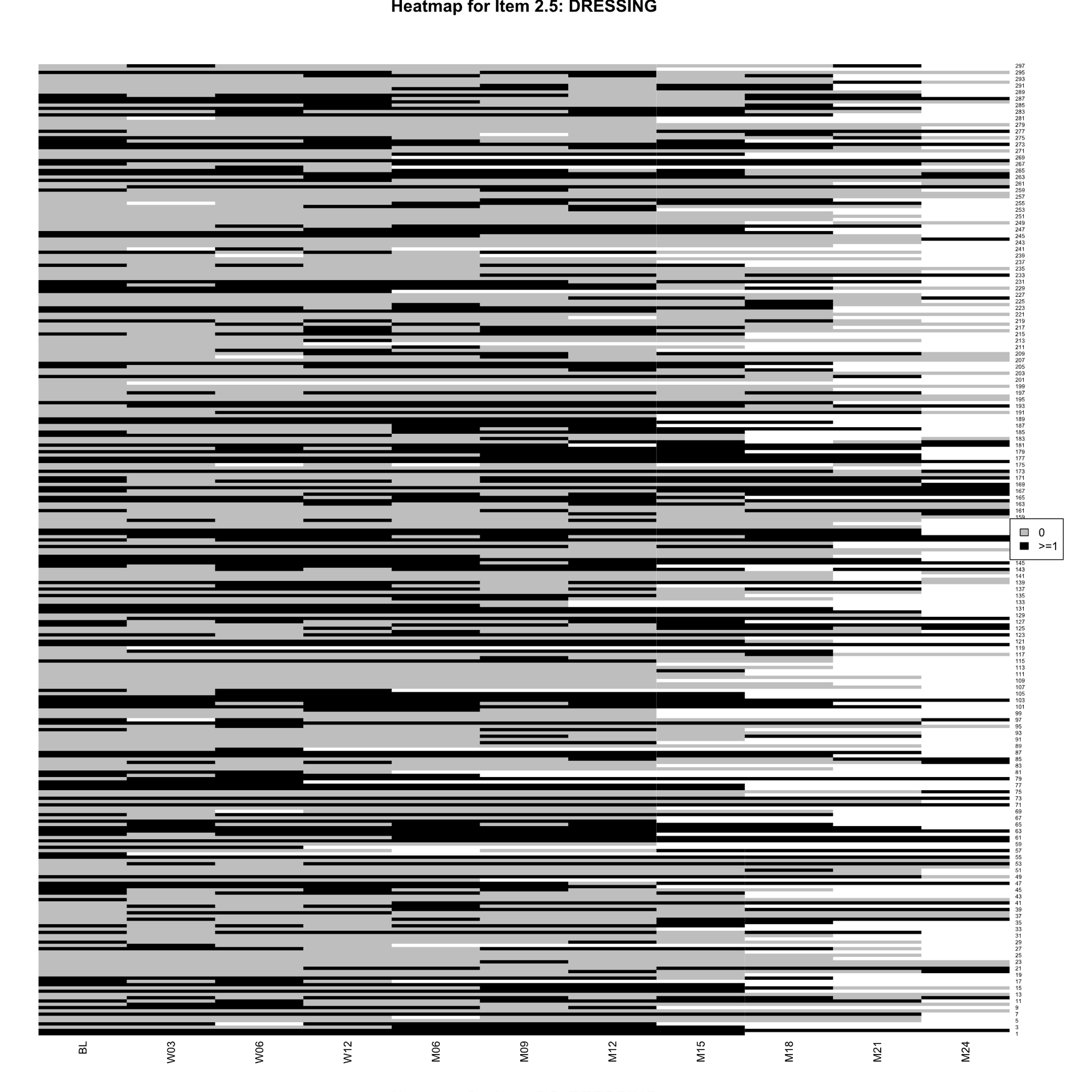

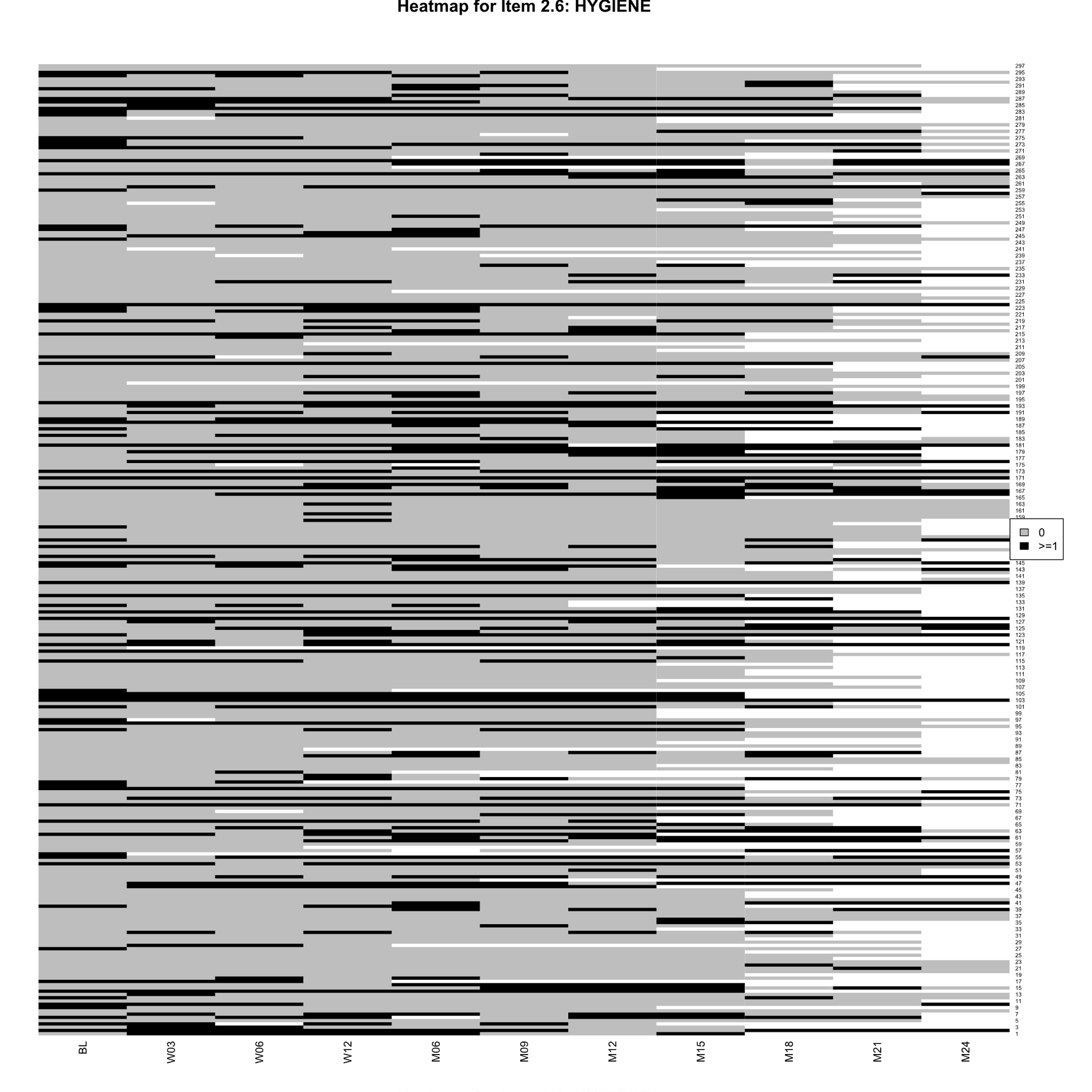

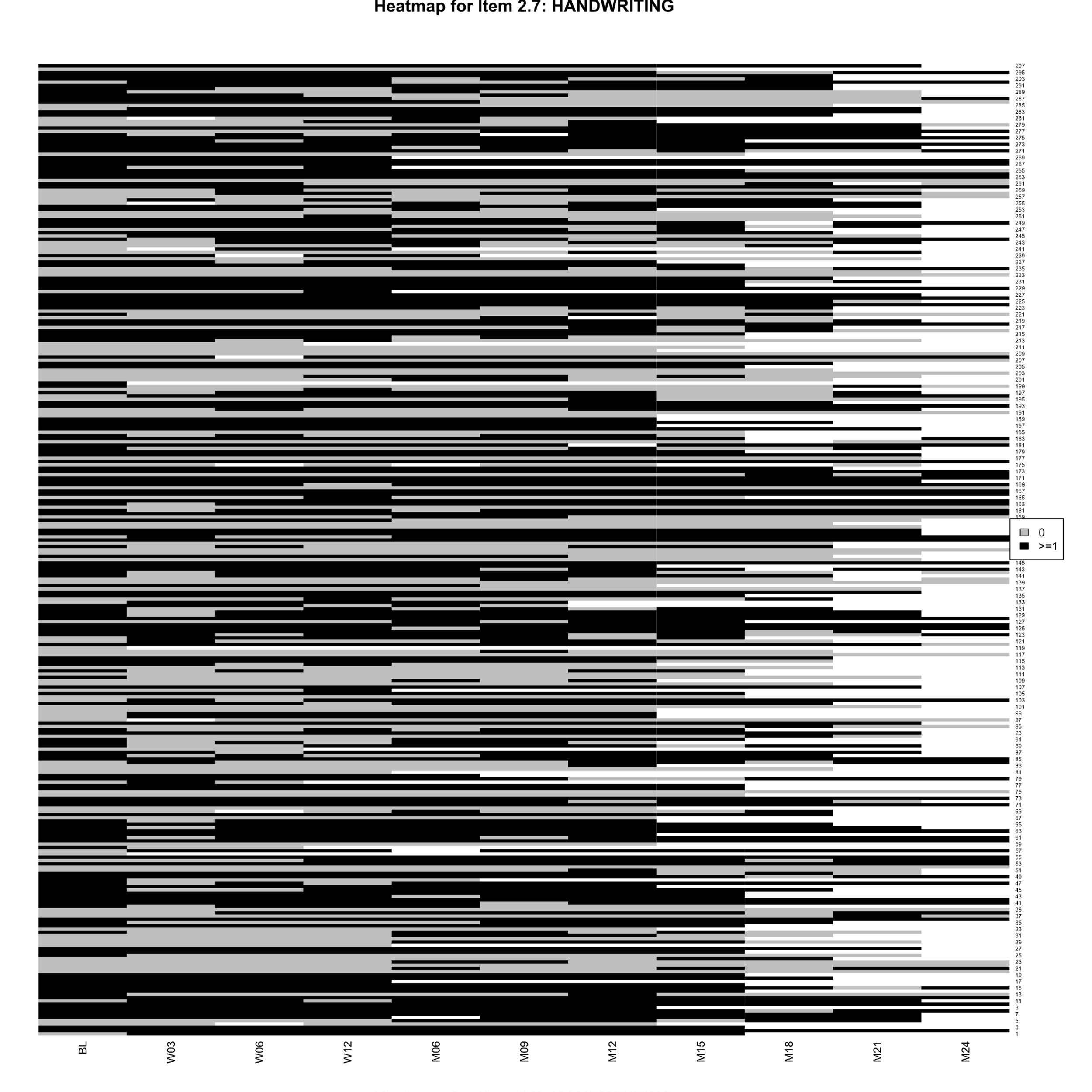

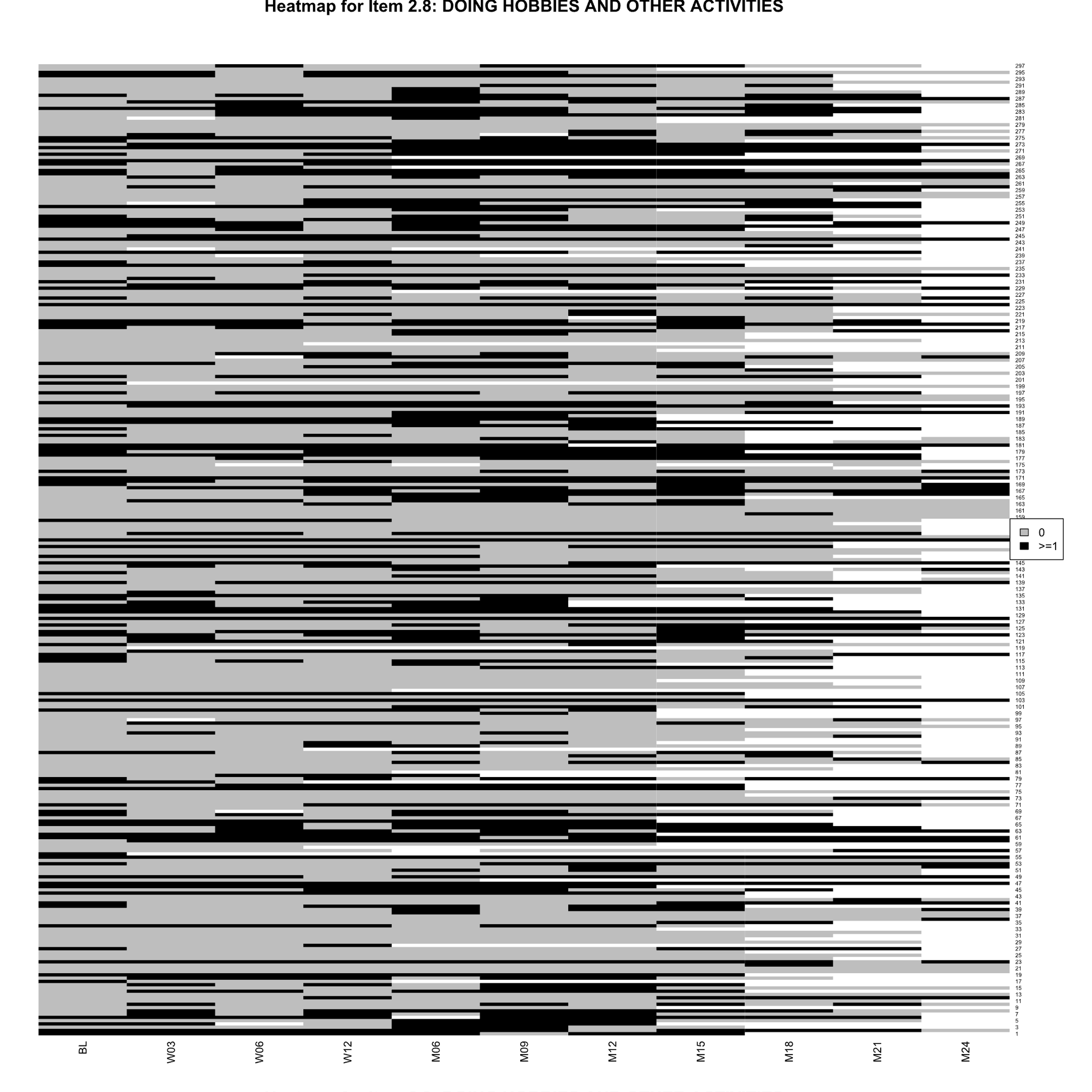

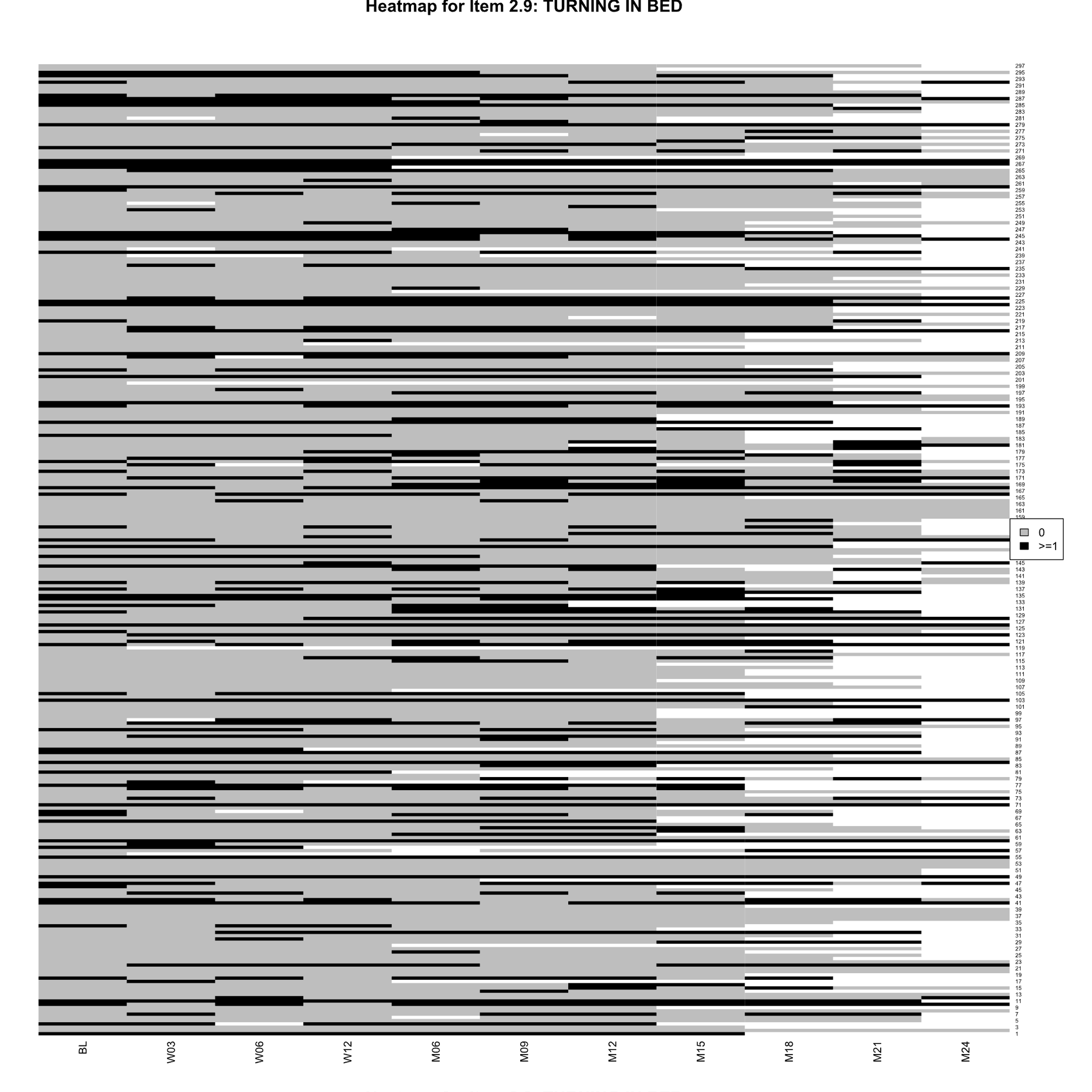

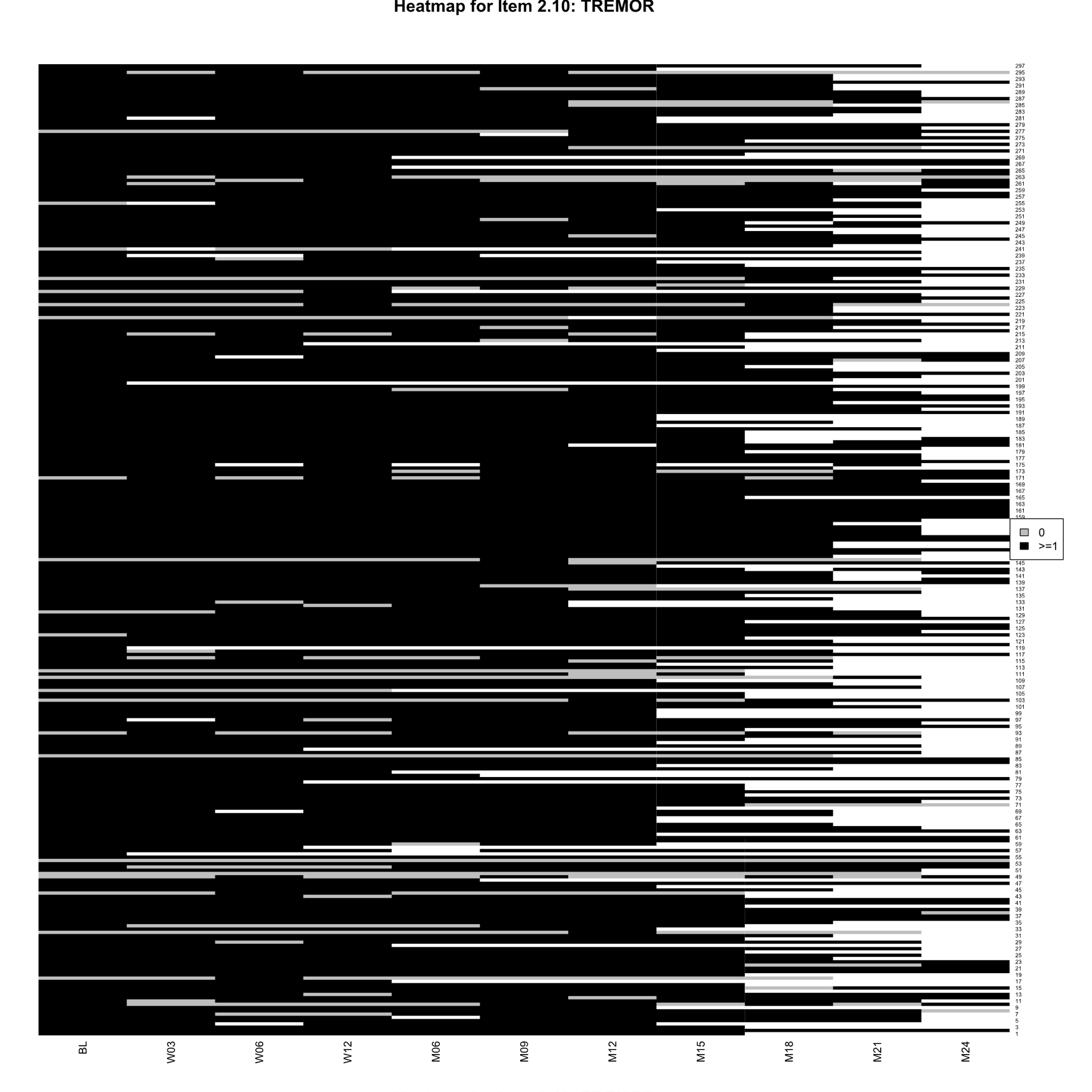

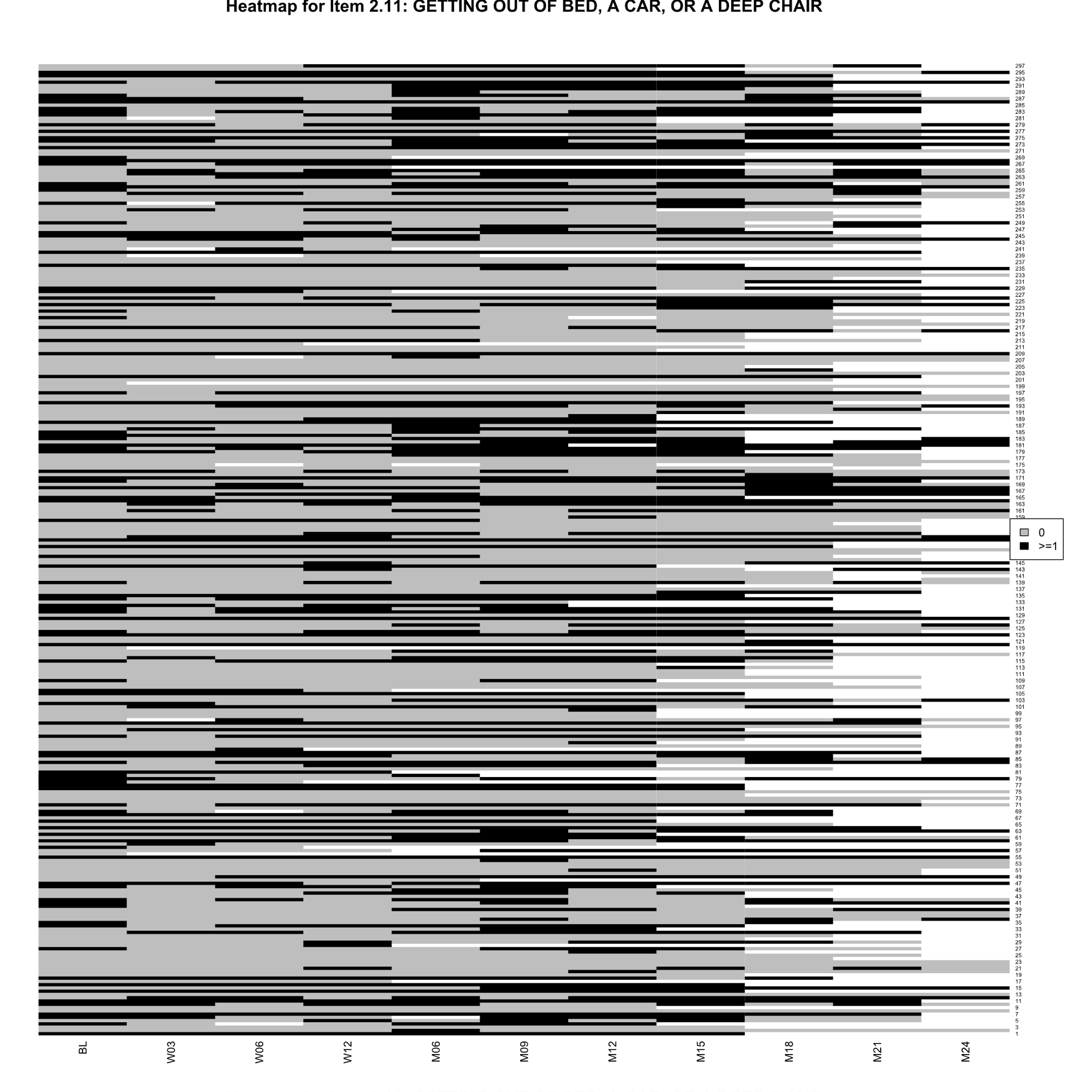

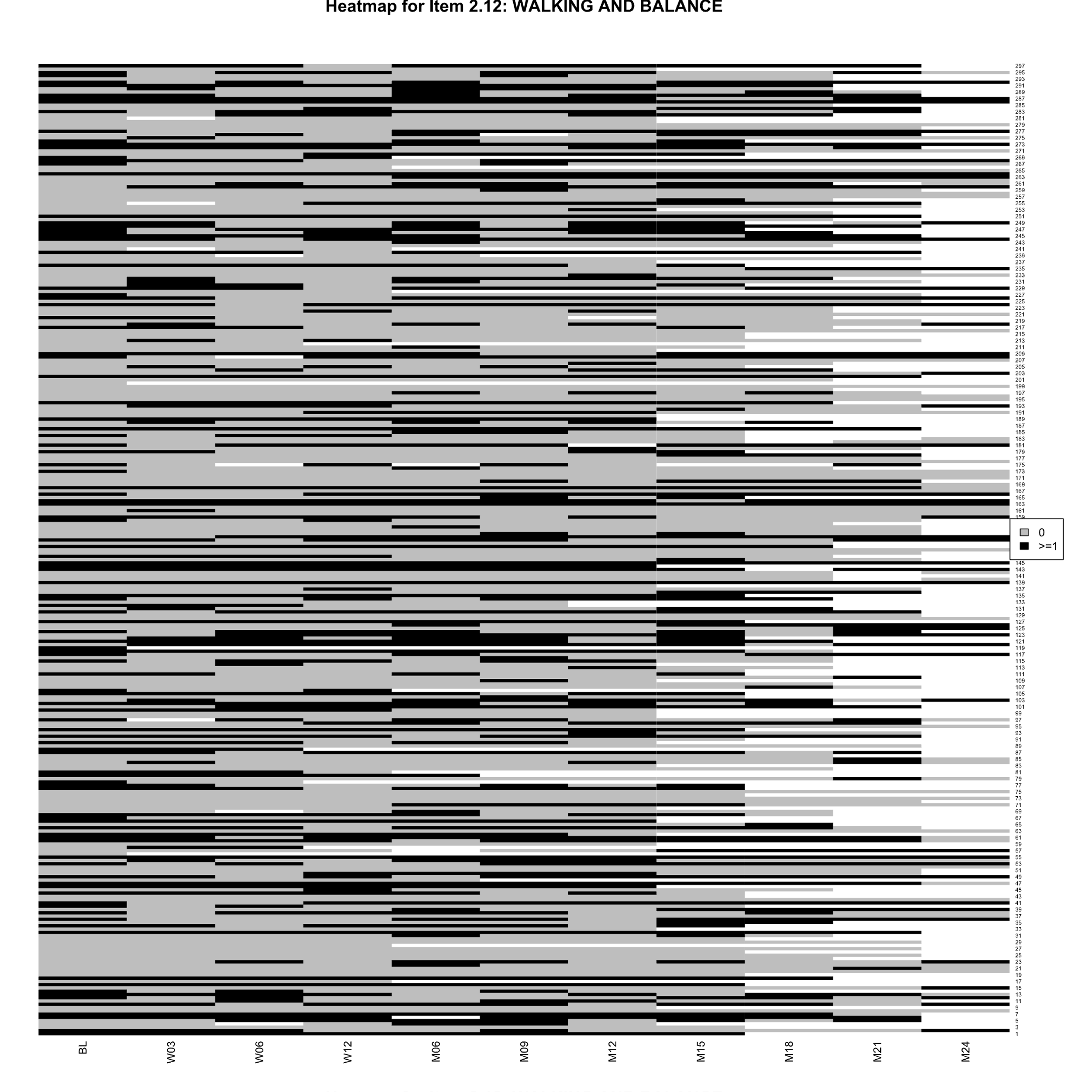

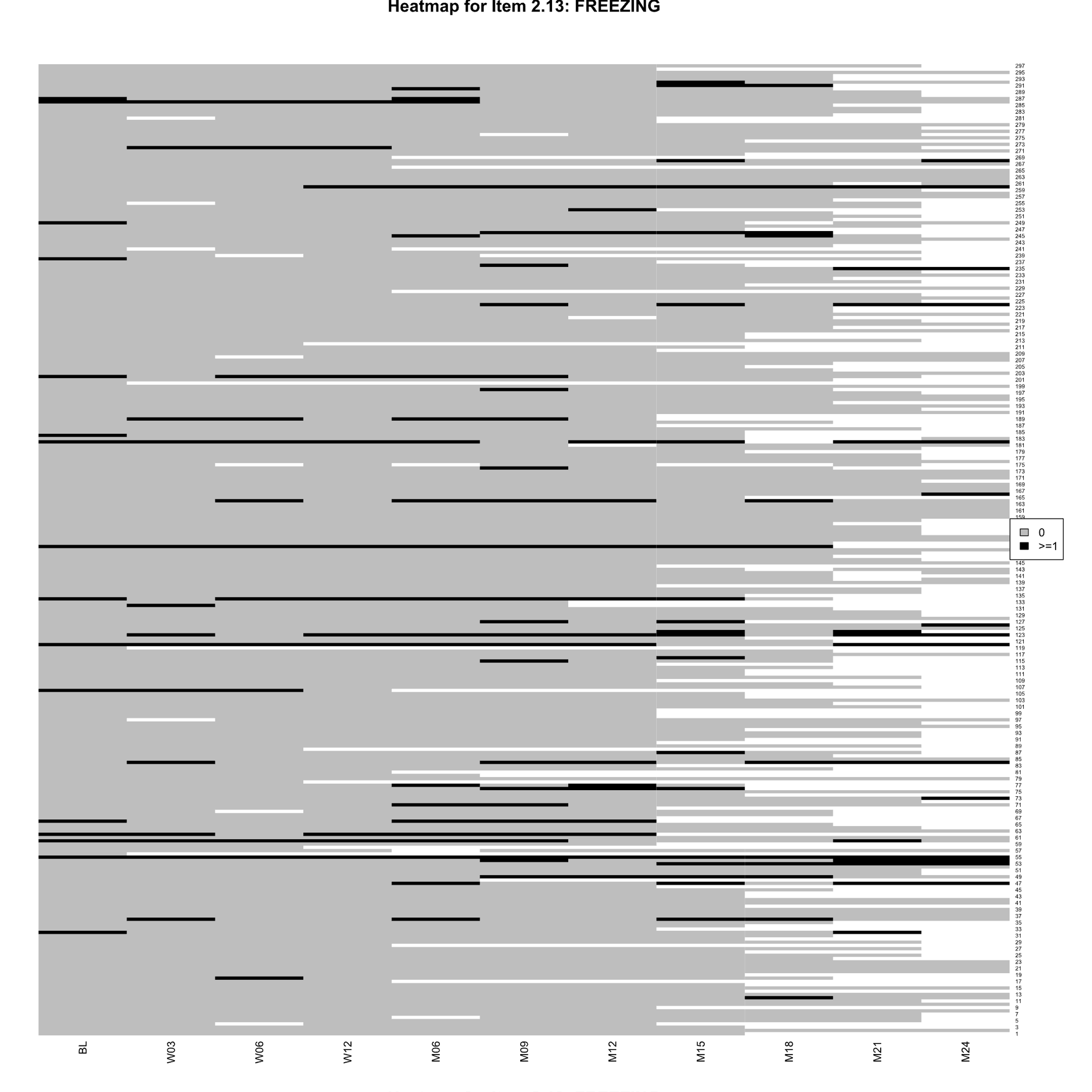

Supplement: Supplement [file NIHMS2108766-supplement-Supplement.docx]
